# Timing and sequence of vaccination against COVID-19 and influenza (TACTIC): a single-blind, placebo-controlled randomized clinical trial

**DOI:** 10.1016/j.lanepe.2023.100628

**Published:** 2023-04-12

**Authors:** Elisabeth A. Dulfer, Büsra Geckin, Esther J.M. Taks, Corine H. GeurtsvanKessel, Helga Dijkstra, Liesbeth van Emst, Christa E. van der Gaast – de Jongh, Djenolan van Mourik, Petra C. Koopmans, Jorge Domínguez-Andrés, Reinout van Crevel, Josephine S. van de Maat, Marien I. de Jonge, Mihai G. Netea

**Affiliations:** a Department of Internal Medicine, Radboud University Medical Center, Nijmegen, the Netherlands; b Radboud Center for Infectious Diseases, Radboud University Medical Center, Nijmegen, the Netherlands; c Laboratory of Viroscience, Erasmus Medical Center, Rotterdam, the Netherlands; d Laboratory of Medical Immunology, Radboud University Medical Center, Nijmegen, the Netherlands; e Department of Biostatistics, Radboud University Medical Center, Nijmegen, the Netherlands; f Department for Immunology and Metabolism, Life and Medical Sciences Institute (LIMES), University of Bonn, Germany

**Keywords:** COVID-19, SARS-CoV2, Infection, Infectious diseases, Vaccines, mRNA, Influenza

## Abstract

**Background:**

Novel mRNA-based vaccines have been used to protect against SARS-CoV-2, especially in vulnerable populations who also receive an annual influenza vaccination. The TACTIC study investigated potential immune interference between the mRNA COVID-19 booster vaccine and the quadrivalent influenza vaccine, and determined if concurrent administration would have effects on safety or immunogenicity.

**Methods:**

TACTIC was a single-blind, placebo-controlled randomized clinical trial conducted at the Radboud University Medical Centre, the Netherlands. Individuals ≥60 years, fully vaccinated against COVID-19 were eligible for participation and randomized into one of four study groups: 1) 0.5 ml influenza vaccination Vaxigrip Tetra followed by 0.3 ml BNT162b2 COVID-19 booster vaccination 21 days later, (2) COVID-19 booster vaccination followed by influenza vaccination, (3) influenza vaccination concurrent with the COVID-19 booster vaccination, and (4) COVID-19 booster vaccination only (reference group). Primary outcome was the geometric mean concentration (GMC) of IgG against the spike (S)-protein of the SARS-CoV-2 virus, 21 days after booster vaccination. We performed a non-inferiority analysis of concurrent administration compared to booster vaccines alone with a predefined non-inferiority margin of −0.3 on the log10-scale.

**Findings:**

154 individuals participated from October, 4, 2021, until November, 5, 2021. Anti-S IgG GMCs for the co-administration and reference group were 1684 BAU/ml and 2435 BAU/ml, respectively. Concurrent vaccination did not meet the criteria for non-inferiority (estimate −0.1791, 95% CI −0.3680 to −0.009831) and antibodies showed significantly lower neutralization capacity compared to the reference group. Reported side-effects were mild and did not differ between study groups.

**Interpretation:**

Concurrent administration of both vaccines is safe, but the quantitative and functional antibody responses were marginally lower compared to booster vaccination alone. Lower protection against COVID-19 with concurrent administration of COVID-19 and influenza vaccination cannot be excluded, although additional larger studies would be required to confirm this.

**Trial registration number:**

EudraCT: 2021-002186-17

**Funding:**

The study was supported by the 10.13039/501100001826ZonMw COVID-19 Programme.

**Research in context**

**Evidence before this study**

During the COVID-19 pandemic, novel mRNA vaccines have successfully been employed to decrease morbidity and mortality worldwide. Booster vaccinations to maintain immunity over a longer time and in the context of new emerging variants were proven to be safe and effective. One of the groups most at risk for severe COVID-19 are older adults and protective efforts have been made to shield this vulnerable population. Before our study started, research had not focused on the potential co-administration of vaccination against the SARS-CoV-2 virus and the influenza virus. A collection of previous research into different vaccines suggests the possibility of interference between some vaccines, but mRNA vaccines had not been studied in this context.

**Added value of this study**

This study did not prove non-inferiority of concurrent administration of the BNT162b2 COVID-19 booster vaccine and the Vaxigrip Tetra influenza vaccine compared to booster vaccination alone, suggesting possible immune interference. To our knowledge, this is the first RCT that investigated immunogenicity of concurrent administration in a representative group of older adults with predefined non-inferiority margins and an additional focus on mucosal antibodies and systemic inflammation.

**Implications of all the available evidence**

The marginally lower serological responses after concurrent vaccination with a COVID-19 booster and an influenza vaccine found in this study are an import aspect to consider in public health policy and future vaccination campaigns aimed at older adults. This is of major importance for the upcoming influenza season, as well as for protection of vulnerable groups against other future pathogens. The findings of this study highlight the need for more research into the potential for immune interference prior to policy decisions concerning simultaneous administration of COVID-19 and influenza vaccines, as well as other vaccine combinations.

## Introduction

The SARS-CoV-2 virus causing coronavirus disease 2019 (COVID-19) has quickly spread worldwide and caused over 6 million deaths since the first case was diagnosed in December 2019.^1^ Novel viral vector vaccines (such as Ad26.COV2.S and ChAdOx1-S) and mRNA-based vaccines (such as BNT162b2 and mRNA-1237) against COVID-19 were introduced in 2021, and showed clear beneficial effects by decreasing morbidity and mortality.^2^^,^^3^ Although considered successful in inducing protection against infection and severe disease, the longevity of this protection has been shown to decline over time. Antibody concentrations in the circulation of vaccinated individuals decreased in a matter of months and new virus variants emerged.^4^^,^^5^ Because of these observations, many countries provided ‘booster shots’ to maintain immunity in the population. The Netherlands started a vaccination campaign with booster shots employing the BNT162b2 vaccine to avoid a 2021 winter surge by the then-dominant Delta variant (B.1.617.2).

The timing of this campaign coincided with the existing annual Dutch vaccination program against the influenza virus, in which more than 3 million persons at risk of severe disease are immunized every autumn. Co-administration of the vaccine against COVID-19 and influenza would provide many logistic advantages, but the combination could theoretically result in both positive and negative responses: ranging from enhanced immunity against both viruses, to inhibition of immune responses to one or both of the viruses due to immune interference. Earlier studies have investigated the co-administration of different live and inactivated vaccines, reporting variable results. In some studies, no effect on immunogenicity of vaccination with live-attenuated influenza vaccines concurrently administered with other common childhood vaccines was measured, while in other studies immune interference was found.^6^, ^7^, ^8^ When administering distinct types of vaccines sequentially, some sequences have been associated with reduced or increased mortality rates.^9^^,^^10^ In contrast to suppression of immunogenicity or protection, it has been suggested that vaccination with an inactivated influenza vaccine could boost the immune response to SARS-CoV-2 by inducing trained immunity.^11^ This same study also indicated that the influenza vaccine could lower systemic inflammation, whereas concerns have been raised about increased inflammation in response to mRNA vaccines.^12^ The long-term inflammatory effects have not been studied in the context of co- or sequential administration of the novel COVID-19 vaccines. Long-term complications resulting from enhanced inflammation could potentially occur and would need to be ruled out.

Vaccine-induced immune interference is difficult to predict and because of their novelty, the immunological and clinical interactions between mRNA vaccines and influenza vaccines had not been studied before. Different sequences of administration may alter their potential effects. To unravel the potential immune interference between these vaccines in terms of immunogenicity and safety, and to establish an optimal vaccination strategy, the TACTIC-study was designed to assess different schemes of administration of these two vaccines. The primary aim of this study was to investigate whether influenza vaccination prior to, after, or combined with COVID-19 vaccination would influence the immune response against SARS-CoV-2 induced by the mRNA vaccine. Investigating this effect is of profound importance for the vaccination strategy in the coming years: both for the upcoming additional booster campaigns against COVID-19,^13^ as well as for the use of novel vaccines in the more distant future.^14^

## Methods

### Study design

**Fig. 1:**
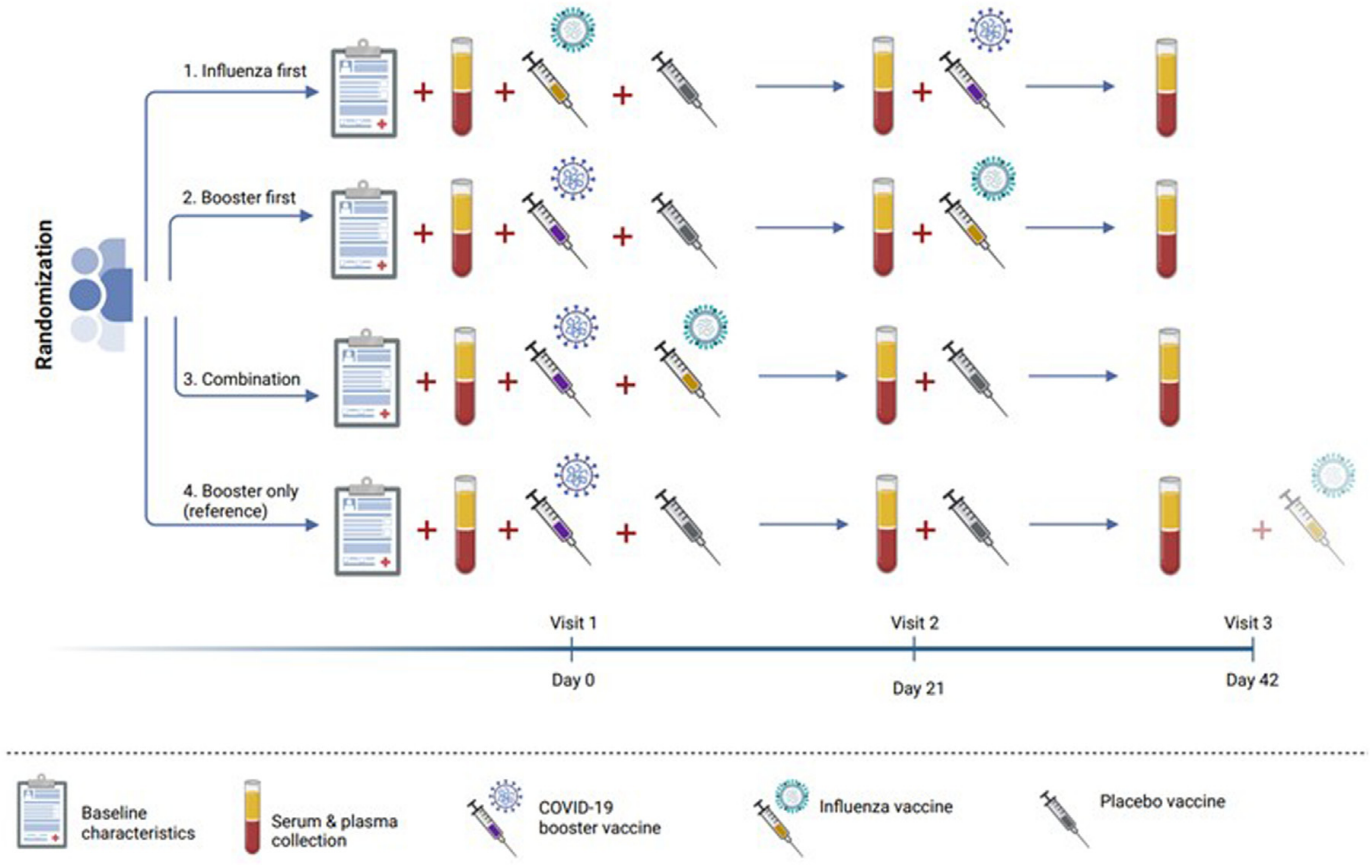
Study design.

The TACTIC study was a single-blind, placebo-controlled randomized clinical proof-of-principle trial conducted at the Radboud university medical center (Radboudumc) in Nijmegen, the Netherlands. The overall aim of the study was to evaluate immunogenicity and safety of combined influenza- and COVID-19 booster vaccinations, investigating four vaccination schemes: (1) influenza vaccination with Vaxigrip Tetra followed by a BNT162b2 COVID-19 booster vaccination 21 days later (hereafter called ‘influenza first’), (2) COVID-19 booster vaccination followed by influenza vaccination 21 days later (‘booster first’), (3) influenza vaccination concurrent with a booster vaccination (‘combination’), and (4) booster vaccination only (‘booster only’). Placebo vaccines were used to prevent the participants from deducing the group they had been placed in (Fig. 1).

This study was performed in accordance with the principles of the Declaration of Helsinki and Good Clinical Practice, as well as the local Radboudumc Research code. Approval was obtained from the competent authority (CCMO; EudraCT number 2021-002186-17) and the medical ethics committee Oost-Nederland (file number 2021-8294). Written informed consent was obtained from all study participants.

### Participants

Participants were recruited by an advertisement in local newspapers, on social media and the Radboudumc research website. Volunteers aged ≥60 years who were fully vaccinated against COVID-19 at least four months prior to study start were eligible for participation (one dose of the Janssen vaccine, two doses AstraZeneca or mRNA vaccine, or one dose AstraZeneca or mRNA vaccine after previous COVID-19 infection). Details of eligibility criteria can be found in the study protocol (attached as supplementary file).

### Randomization and masking

Castor Electronic Data Capture system (Castor EDC) randomized participants to one of the four study groups, giving equal weight to all groups and using variable block size (4, 8, 12). Participants were blinded to their group allocation by using identical syringes for all vaccines to minimize influence on reported adverse events. Trial personnel were not blinded.

### Procedures

The study encompassed three study visits, each 21 days apart (see Fig. 1). During the first study visit, participants gave informed consent and baseline characteristics were recorded. Vaccines were administered intramuscularly in the upper arm during visit 1 and 2 and participants were observed 15 min after vaccination. We used the recommended dose of 0.5 ml Vaxigrip Tetra and 0.3 ml BNT162b2 COVID-19 booster vaccine, as well as 0.5 ml sterile NaCl 0.9% as a placebo. In the case of two concurrently administered vaccines, two different injection sites in opposite arms were used when medically possible. Plasma, serum and mucosal lining fluid (MLF) samples were obtained during all three visits (T1 – T3). MLF was collected using Nasosorption™ FXi nasal sampling devices (Hunt Developments, UK). Participants used paper diaries to report any adverse event or possible side-effects for 14 days after each vaccination and assessed severity on a 5-point Likert-scale (‘none’ to ‘extreme’). The side-effects listed in the diary were based on the most common side-effects associated with the used vaccines.

### Outcomes

#### Primary endpoint

Geometric mean concentration of IgG responses against the spike (S)-protein of the SARS-CoV-2 virus in plasma, at 21 days after booster vaccination.

#### Secondary endpoints

- IgA responses against S-protein and IgA and IgG responses against receptor binding domain (RBD) in plasma at baseline, 21 days after each vaccination;
- IgA and IgG responses against the nucleocapsid (N)-protein to control for infection during the study;
- Seroconversion of IgG to S-protein at day 21 after the COVID-19 booster vaccine (defined as a change from seronegative at baseline (T1) to seropositive or a ≥four-fold increase);
- Neutralization of the ancestral SARS-CoV-2 Wuhan, B.1.617.2 (delta) and B.1.1.529 (omicron) variants, at 42 days after first study vaccination round;
- IgA and IgG responses against S- and N- protein in mucosal lining fluid at baseline and 21 days after each vaccination;
- Hemagglutinin inhibition titers in serum at 21 days after influenza vaccination;
- Serious adverse events (SAEs) and other adverse events (AE);
- Local reactions at injection site or systemic reactions after vaccination.

#### Exploratory endpoint

Assessment of systemic inflammation by targeted proteome analysis.

### Statistical analysis

#### Sample size calculation

The required sample size to evaluate non-inferiority of the primary endpoint had been calculated based on geometric mean IgG titers after vaccination with the BNT152b2 vaccine. The aim was to include 35 participants per intervention group, providing 90% power to evaluate non-inferiority of ‘influenza first’, ‘booster first’ and ‘concomitant influenza- and COVID-19 booster vaccine’, compared to a COVID-19 booster alone, considering an estimated means of −0.3 on the log10-scale as a non-inferiority margin. It must be noted that this was a conservative calculation, since the expected IgG titer variability (SD) after booster vaccination was likely to be lower than after primary vaccination, but no evidence was available at the time.

#### Comparative analyses

Statistical analyses were performed using GraphPad prism version 8, IBM SPSS Statistics for Windows, Version 26, R version 4.1.3 and SAS version 9.4. Analyses were performed according to the intention-to-treat principle. As no participants switched to another study group (intentionally or accidentally), these analyses equal a per-protocol population.

Baseline and safety variables were compared between participants in the different study groups, and differences were statistically tested using χ^2^ tests, Fisher's exact tests (because of small numbers) or t-tests for independent samples as appropriate. For safety results, relative risks were calculated. Non-inferiority of the ‘concurrent administration’ was evaluated, comparing the anti-S IgG levels of that group with the reference group who received a booster vaccination only. We performed a linear mixed models analysis using Proc Mixed Model in SAS with the log transformed anti-S IgG concentrations at day 21 as outcome and group as a fixed factor. We used the Kenward-Roger method for computing the denominator degrees of freedom for the tests of fixed effects. Least squares means estimates of all groups were compared with the reference group (COVID-19 booster only) and a Dunnett correction was used to adjust for multiple testing (overall alpha = 5%). For the interpretation of non-inferiority, Dunnett adjusted confidence intervals of the differences in least square means are presented in the results. If the lower limit of the adjusted confidence interval lies above the predefined non-inferiority margin of −0.3 on the log10-scale, we would conclude that the result of the corresponding group is non-inferior to the reference group.

We performed a sensitivity analysis of the primary endpoint, adjusting for differences in log transformed baseline IgG against SARS-CoV-2. As subsequent sensitivity analyses, we also adjusted for pneumococcal vaccine at baseline and baseline levels of anti-N IgG, as well as excluding participants who reported prior COVID-19. To conclude the non-inferiority analyses, we combined the ‘booster first’ with the ‘booster only’ group to create a larger reference group. We checked the assumption of normality of residuals graphically and the residuals were normally distributed.

Antibody levels against the SARS-CoV-2 S-, RBD- and N-protein over the course of the study were measured and reported as geometric mean concentrations over time. Anti-N levels were measured to determine if any of the participants contracted a SARS-CoV-2 infection during the study. Qualitative serology titers for the reference group and different vaccination schemes were compared using Mann–Whitney U-tests. Mucosal anti-S IgG antibodies were correlated to antibodies from plasma using Pearson correlation. Protein measurements were denoted as normalized protein expression values (NPX) and analyzed by principal component analysis, including all four study groups. Participants from which one or more proteins could not be measured are not included in this PCA analysis. Wilcoxon paired signed-rank test was used to compare NPX values at 42 days after initial study vaccination to baseline, for each study group separately. Benjamini-Hochberg adjustment was used to correct the proteomics data for multiple testing. A total of 44 out of 92 measured proteins were detected in at least 70% of the plasma samples and were included in the analyses (see supplementary methods S1).

Effect estimates were reported with 95% confidence intervals. All statistical tests were performed in a two-sided manner and a p-value <0.05 was considered statistically significant. Given the set-up of the trial, the relative short study period and the use of established investigational products, no data monitoring committee was employed.

### Laboratory analyses

Blood samples were obtained from the cubital vein and stored at −80 °C prior to analysis. Mucosal lining fluid absorption strips were placed back into protective plastic tubes after sampling and stored at −20 °C until further processing.

#### Serology and mucosal antibodies

To measure the levels of antibodies against RBD and Spike protein, a fluorescent-bead-based multiplex immunoassay (MIA) was developed as previously described by Fröberg et al., with some slight modifications.^15^ The first international standard for anti-SARS-CoV-2 immunoglobulin, (20/136, NIBSC), was used to create standard curves. Next to this, four different samples from PCR-confirmed COVID-19 patients were used as quality control samples. The stabilized pre-fusion conformation of the ectodomain of the S-protein (D614G mutant) and the RBD-protein, both purchased from ExcellGene (Monthey, Switzerland), were each coupled to beads or microspheres with distinct fluorescence excitation and emission spectra. Serum samples were diluted in assay buffer (SM01/1%BSA) and incubated for 45 min with the antigen-coated microspheres. Following incubation, the microspheres were washed three times and incubated with phycoerythrin-conjugated goat anti-human, IgG. The data were acquired on the Luminex FlexMap3D System. Validation of the detection antibodies was obtained from a recent publication using the same antibodies and the same assay,^16^ and specificity was checked using rabbit anti-SARS SIA-ST serum. Mean fluorescent intensities (MFI) were converted to binding antibody units (BAU/ml) by interpolation from a log-5PL-parameter logistic standard curve and log–log axis transformation, using Bioplex Manager 6.2 (Bio-Rad Laboratories) software and exported to R-studio.

#### Plaque reduction neutralization assays

Serum samples were tested for the presence of neutralizing antibodies against ancestral SARSCoV-2, Delta and Omicron (BA.1) variants in a plaque reduction neutralization test (PRNT) as previously described.^17^, ^18^, ^19^ Viruses were cultured from clinical specimen and were confirmed by next-generation sequencing: D614G (ancestral, GISAID: hCov19/Netherlands/ZH-EMC-2498), B.1.617.2 (Delta, GISAID: hCoV-19/Netherlands/NB-MVDCWGS2201159/2022), and B.1.1.529 (Omicron BA.1, GISAID: hCoV-19/Netherlands/LISQD-01032/2022).

The human airway Calu-3 cell line (ATCC HTB-55) was used to grow virus stocks and for PRNT. Calu-3 cells were cultured in OptiMEM (Gibco) supplemented with Glutamax, penicillin (100 IU/ml), streptomycin (100 IU/ml), and 10% fetal bovine serum (FBS). In short, heat-inactivated sera were diluted two-fold in OptiMEM without FBS starting at a 1:10 dilution, or in the case of a S1-specific antibody level >2500 BAU/mL, starting at 1:80 in 60 μL. 400 PFU of each SARS-CoV-2 variant in 60 μL OptiMEM medium was added to diluted sera and incubated at 37 °C for 1 h. Antibody-virus mix was transferred onto Calu-3 cells and incubated at 37 °C for 8 h. Cells were fixed in PFA and stained with polyclonal rabbit anti-SARS-CoV-2 nucleocapsid antibody (Sino Biological) and a secondary peroxidaselabeled goat-anti rabbit IgG antibody (Dako). Signal was developed with precipitate-forming 3,3′,5,5′-tetramethylbenzidine substrate (TrueBlue; Kirkegaard & Perry Laboratories) and the number of plaques per well was counted with an ImmunoSpot Image Analyzer (CTL Europe GmbH). The 50% reduction titer (PRNT50) was estimated by calculating the proportionate distance between two dilutions from which the endpoint titer was calculated. Infection controls (no sera) and positive serum control (Nanogram® 100 mg/ml, Sanquin) were included on each plate. A PRNT50 value one dilution step (PRNT50 = 10) lower than the lowest dilution was attributed to samples with no detectable neutralizing antibodies.

#### Hemagglutinin inhibition assays

Hemagglutination inhibition (HAI) assays were performed following standard protocols.^20^ Briefly, treated serum samples were serially diluted two-fold and mixed with virus stock (25 μL) containing 4 hemagglutinating units, which incubated for 30 min at 37 °C. Turkey erythrocyte solution (25 μL, 1%) was added and after 1 h incubation at 4 °C inhibition patterns were recorded. Titers were expressed as the value of the highest serum dilution that gave complete inhibition of agglutination.

#### Proteomics

Plasma proteins were measured using the Olink Inflammation panel by Olink Proteomics (Uppsala, Sweden).

### Protocol amendments

The vector vaccine Ad26.COV2.s produced by Janssen was initially also included in the study, but when it became apparent this would not be used in the Dutch booster campaign, it was removed from the protocol. New emerging SARS-CoV-2 variants were added to the analysis (B.1.617.2 and B.1.1.529). Before the study started, the timepoint for primary analysis was altered from 21 days after last vaccination to 21 days after booster vaccination, as we considered this more relevant for our research question. The final study protocol can be found in a supplementary file.

### Role of the funding source

The study was supported by the COVID-19 program of the Dutch Organization for Scientific Research (10.13039/501100001826ZonMw). The funder had no role in study design, data collection, data analysis, data interpretation, or writing of the report.

## Results

### Study population

**Supplementary Fig. S1:**
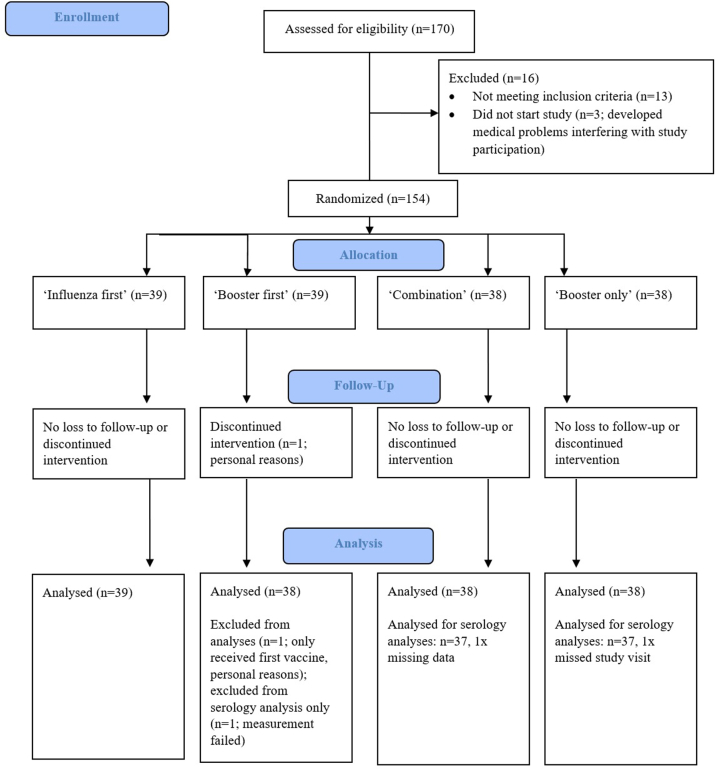


We included 154 individuals between October 4, 2021 and November 5, 2021. 88 participants were male (56%) and the median age of volunteers was 66 years (see Table 1). 153 (99%) participants completed the study and received the intended vaccines in the predetermined order, according to their respective randomization (see flow diagram in supplementary materials, supplementary Fig. S1). The majority (100/154, 65.3%) had received previous SARS-CoV-2 vaccines from Pfizer/BioNTech and only 3 individuals (2%) had experienced COVID-19 before study start. The average time between the last primary SARS-CoV-2 vaccination dose and the study start was four to five months.

**Table 1:** Baseline characteristics.

	Overall (N = 154)	1) Influenza first (N = 39)	2) Booster first (N = 39)	3) Combination (N = 38)	4) Booster only (N = 38)
**Demographics**					
Age, years [median, (IQR)]	66.0 (64–72)	66.0 (64–73)	66.0 (62–71)	67.5 (64–74)	65.5 (63–71)
Male sex	88 (57.1%)	19 (48.7%)	21 (53.8%)	25 (65.8%)	23 (60.5%)
Actively smoking	88 (57.1%)	25 (64.1%)	19 (48.7%)	19 (50.0%)	25 (65.8%)
BMI [mean, (SD)]	25.8 (4.3)	25.4 (4.0)	25.9 (4.0)	25.7 (3.5)	26.4 (5.6)
**SARS-CoV-2**					
History of COVID-19	3 (1.9%)	1 (2.6%)	0 (0.0%)	0 (0.0%)	2 (5.3%)
Pfizer vaccine previously	101 (65.6%)	24 (61.5%)	28 (71.8%)	27 (71.1%)	22 (57.9%)
AstraZeneca vaccine previously	52 (33.8%)	15 (38.5%)	11 (28.2%)	11 (28.9%)	15 (39.5%)
Moderna vaccine previously	1 (0.6%)	0 (0.0%)	0 (0.0%)	0 (0.0%)	1 (2.6%)
Days since last vaccination [mean, (SD)]	146 (33)	142 (23)	154 (44)	145 (32)	143 (27)
**Vaccination status**					
History of BCG vaccination	51 (33.1%)	15 (38.5%)	12 (30.8%)	16 (42.1%)	8 (21.1%)
History of pneumococcal vaccine	17 (11.0%)	5 (12.8%)	0 (0.0%)	7 (18.4%)	5 (13.2%)
Influenza vaccine in season ‘20/’21	138 (89.6%)	35 (89.7%)	34 (7.2%)	35 (92.1%)	34 (89.5%)

### IgG responses against SARS-CoV-2 S-protein

The reference group receiving only a COVID-19 booster vaccination acquired the highest GMC of 2542.2 BAU/ml (binding antibody units) at 21 days after COVID-19 booster vaccination; the combination group with concurrent vaccination showed a lower response with a GMC of 1683.6 BAU/ml. GMCs for ‘influenza first’ and ‘booster first’ were 2347.9 and 2136.8 BAU/ml, respectively. Concurrent vaccination did not meet criteria for non-inferiority (estimate −0.1791, 95% CI −0.3680 to −0.009831). Sensitivity analyses correcting for multiple variables as explained previously, did not change this (supplementary Table S1). When comparing concurrent vaccination to the merged reference group of ‘booster only’ and ‘booster first’, this did show non-inferiority (estimate −0.1165, 95% CI −0.2507 to 0.01767). The vaccination schemes incorporating 21 days in between both vaccines were both non-inferior when compared to vaccination with the booster vaccine alone.

**Fig. 2:**
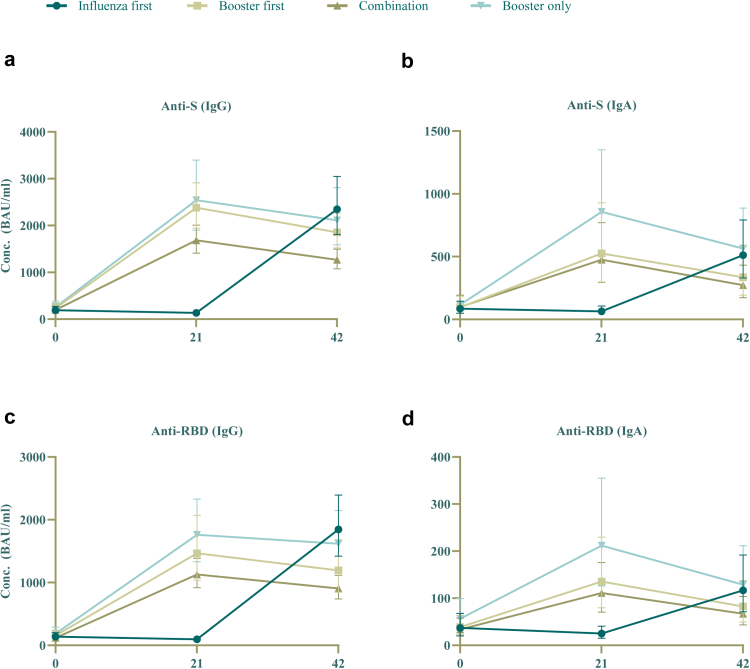
Geometric mean concentrations (with 95% error bars) of IgA and IgG antibodies against S-protein and RBD over the course of the study. a: anti-S IgG; b: anti-S IgA; c: anti-RBD IgG; d: anti-RBD IgA.

Anti-S antibody concentrations after booster vaccination initially rose in the first 3 weeks but started declining within 42 days of vaccination, at a similar rate across all four groups (see Fig. 2). GMCs at baseline and 21 days after booster vaccination can be found in Table 2.

**Table 2:** Geometric mean concentrations (GMCs) of anti-S IgG at baseline and at 21 days after booster vaccination.

	IgG against S-protein (BAU/ml)
**Influenza first (N = 39)**	
Baseline	190.4
Day 21 after booster	2347.9
**Booster first**	
Baseline (N = 39)	225.1
Day 21 (N = 38)	2136.8
**Combination (N = 37)**	
Baseline	199.0
Day 21	1683.6
**Booster only**	
Baseline (N = 38)	258.2
Day 21 (N = 37)	2542.8

Sensitivity analysis adjusting for differences in baseline IgG and previous pneumococcal vaccination showed concurrent vaccination still did not meet non-inferiority criteria when compared to vaccination with a booster only, with respect to IgG response (estimate −0.1391, 95% CI −0.3034 to 0.02510).

### IgA and IgG responses against RBD-, S- and N-protein

Over the course of the study, IgA and IgG anti-RBD antibodies and IgA anti-S levels amongst all groups showed the same trend of an initial rise and subsequent decline (see Fig. 2; b–f; supplementary Fig. S3 for individual data points), in similar fashion to anti-S IgG. Corresponding GMCs can be found in supplementary Table S2. No relevant differences in anti-N antibodies compared to baseline were measured, indicating that none of the participants were infected with SARS-CoV-2 during the study (see supplementary Fig. S2).

Antibodies found in mucosal lining fluid showed patterns comparable to those found in plasma (r = 0.476, p=<0.01; see supplementary Fig. S4, supplementary Fig. S5 for individual data points).

### Seroconversion of IgG against S-protein at 21 days after booster vaccination

All participants had been vaccinated against SARS-CoV-2 before the start of the study and baseline results showed the presence of anti-S IgG antibodies. Across all study groups, a large majority showed seroconversion at 21 days after booster vaccination: 35/39 (89.7%) in the ‘influenza first’ group, 27/37 (73%) in ‘booster first’, 27/37 (75%) in the ‘combination group’, and 31/37 (83 0.8%) in ‘booster only’. There were no significant differences between groups (p = 0.1747).

### Virus neutralization

**Fig. 3:**
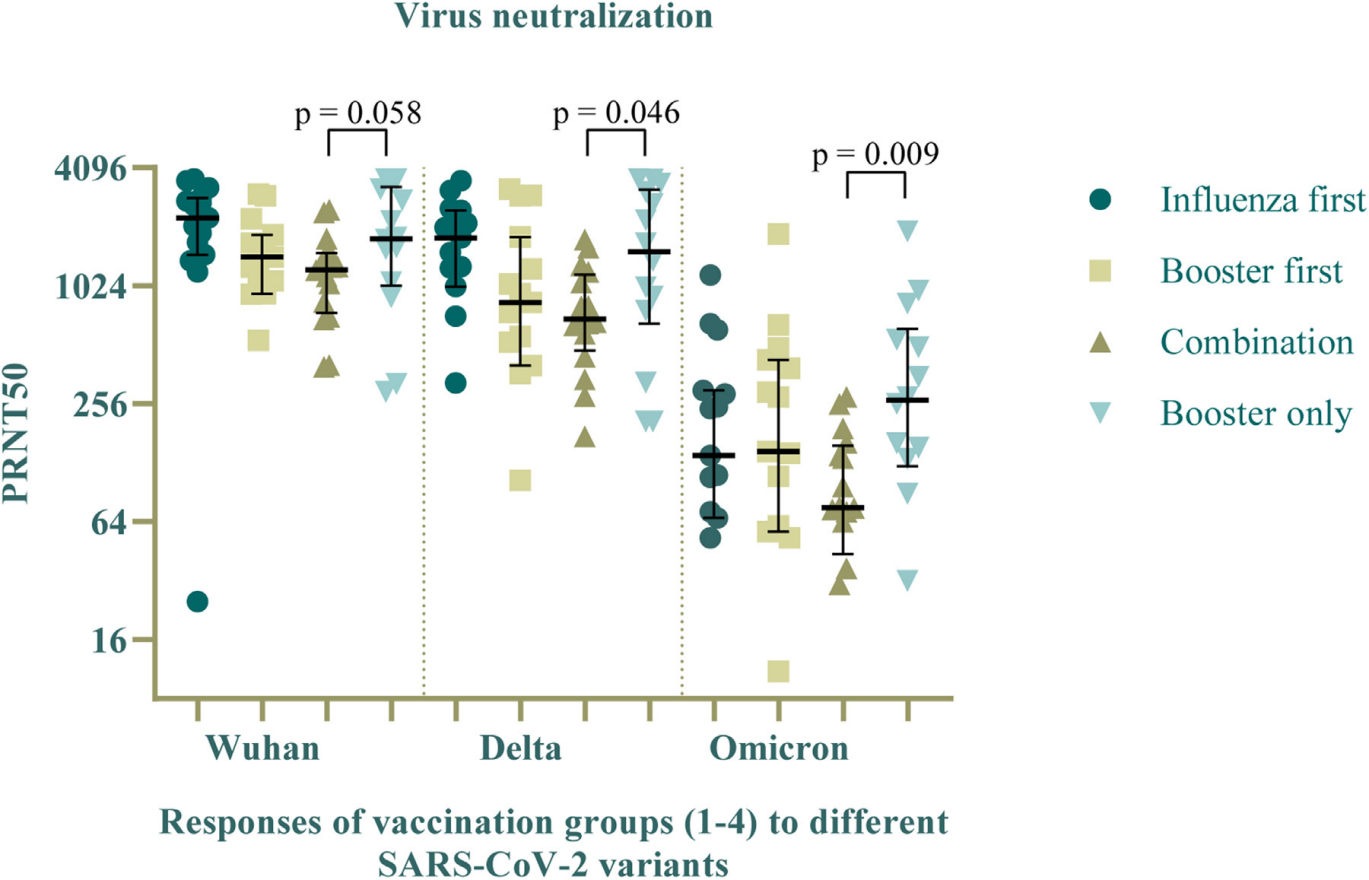
50% plaque-reducing neutralization titers (PRNT-50) for the D614G, delta and omicron variant of the SARS-CoV-2 virus, compared between all groups at 42 days after first study vaccinations (Visit 3).

The neutralizing capacity of the induced antibodies showed comparable plaque-reducing neutralization titers for the original and delta-variant of the SARS-CoV-2 virus, but markedly lower effectivity against the omicron-variant (see Fig. 3). The ‘combination group’ showed significantly lower virus neutralizing capacity than the reference group, as a higher antibody concentration was needed to neutralize 50% of the viral plaque (log2 titers 1:690.5 versus 1:1530, p = 0.0463 for Delta; 1:75.5 versus 1:266.5, p = 0.0093 for Omicron). Neutralization results were not statistically different between the reference group and the consecutive vaccination schemes.

### Hemagglutinin inhibition assays

**Fig. 4:**
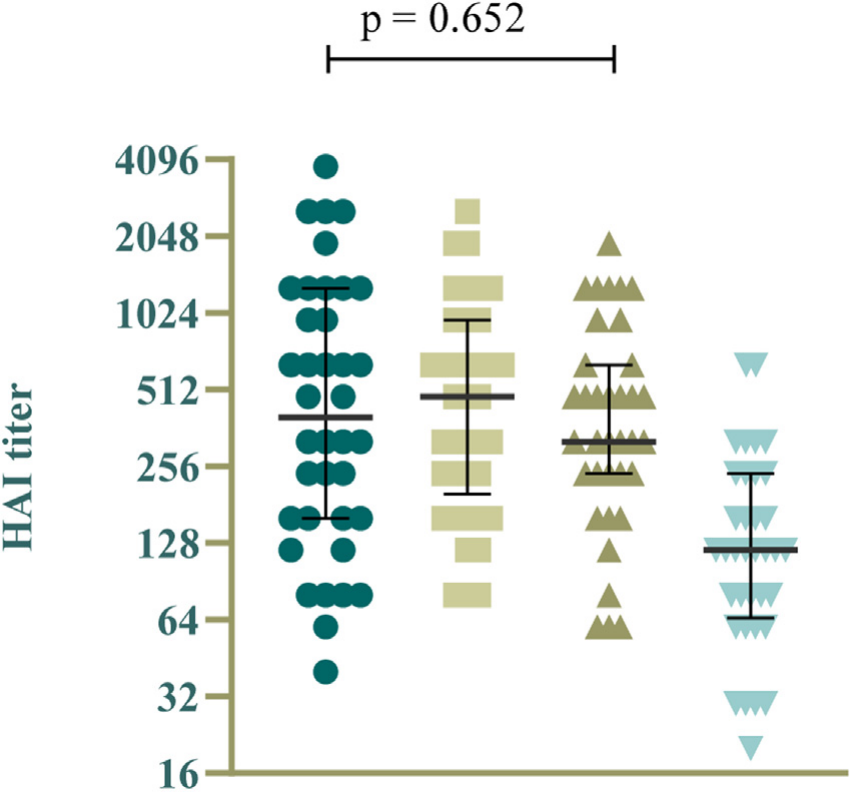
Hemagglutinin inhibition (HAI) titers against the H1N1pdm influenza virus at 21 days after influenza vaccination (groups ‘influenza first’, ‘booster first’, ‘combination’ and 'booster only').

The HAI results show the induction of antibodies against influenza for all three groups who received an influenza vaccine (Fig. 4), 21 days after influenza vaccination. No significant difference in titers was found between these groups, notably not between the ’combination’ group and the ‘influenza first’ group, who, at the time, had only received an influenza vaccination.

### Systemic inflammation after vaccinations

**Fig. 5:**
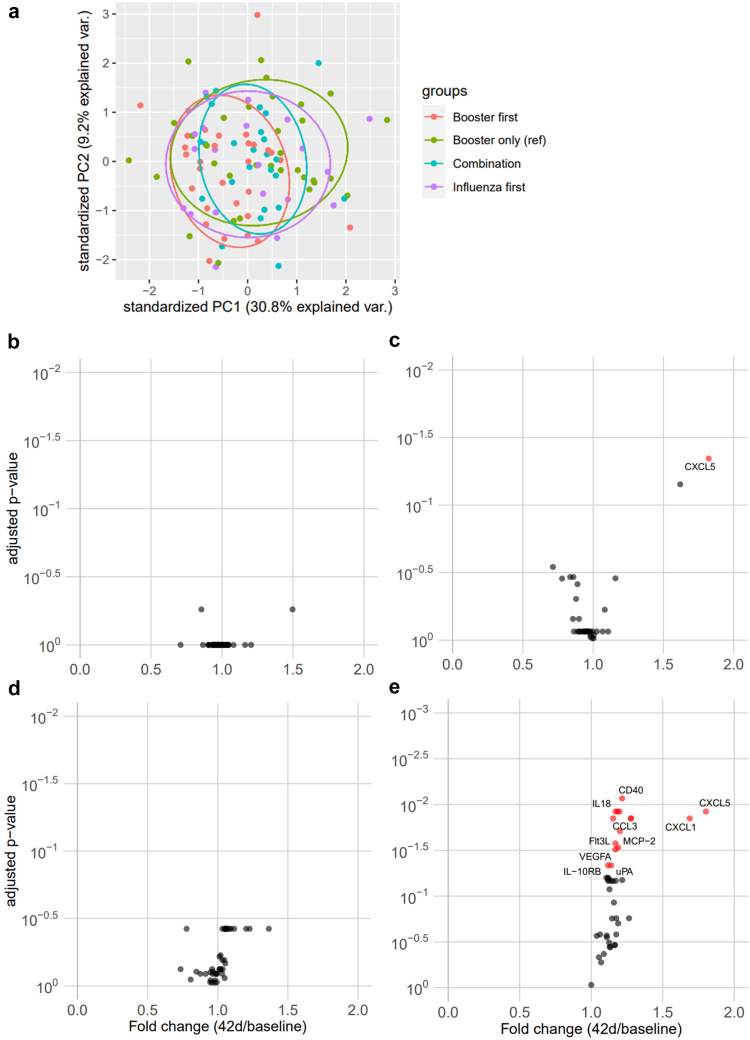
a): Principal component analysis of plasma proteins at 42 days after first study vaccine. (b–e): Volcano plot with fold changes of proteins in all four groups, 42 days after first study vaccine compared to baseline. b): Booster first. c): Influenza first. d): Combination. e): booster only.

Principal component analysis confirmed that our four study groups were generally comparable without extreme outliers and showed none of the vaccination schemes radically changed a group (Fig. 5a). Specific comparisons of the effects of the mRNA booster vaccine showed upregulated inflammatory proteins after booster vaccination, most pronounced at 42 days after vaccination (Fig. 5b–e).

### Safety of the vaccination schemes

One serious adverse event occurred during the study (acute cholecystectomy in the ‘booster first’ group) and has been assessed to be unrelated to any study procedure. After recovery, the participant took part in the final study visit.

### Local and systemic side-effects

**Supplementary Fig. S6:**
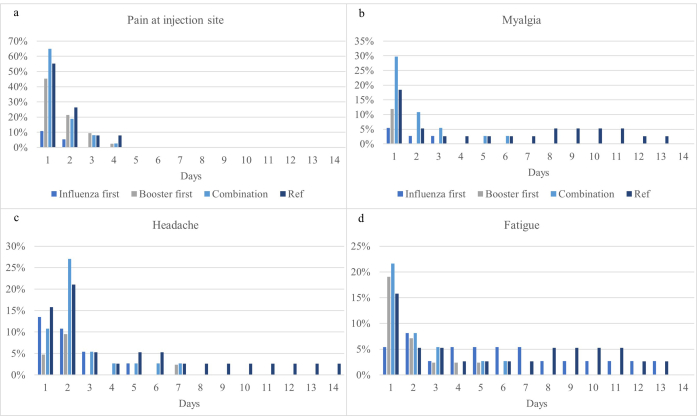


The reported side-effects were considered mild and more than 75% of symptoms resolved spontaneously after 2–3 days in all study groups. No participants sought medical advice for their symptoms and no unexpected side-effects occurred. An overview of the side-effects per group can be found in supplementary Table S3. The most commonly reported side-effects after influenza vaccination were redness and pain at injection site, headache and fatigue. After COVID-19 booster vaccination, pain at injection site, myalgia and headache were the most prominent (see supplementary Fig. S6). Relative risks for participants in the ‘combination’ group compared to the reference group (‘booster only’) did not differ for any of the side-effects (supplementary Table S4).

## Discussion

This study presents the results from the TACTIC trial, designed to investigate the impact of co-administration of an mRNA COVID-19 booster vaccine and an influenza vaccine on the vaccine safety and antibody responses. Based on our results, we cannot exclude non-inferiority of concurrent administration of a COVID-19 booster vaccine and an influenza vaccine, compared to COVID-19 booster vaccine only. Both quantity and functionality of the antibody response against SARS-CoV-2 was diminished when compared to receiving a booster vaccine alone or administration regimens allowing 3 weeks between the vaccines. In regards to safety of concurrent administration, we found no additional or more severe adverse events when compared to sequential administration.

The most important observation of this study is that simultaneous administration of a COVID-19 booster and an influenza vaccination results in a lower serological response against SARS-CoV-2. The predefined non-inferiority criteria for comparing antibody concentrations between the simultaneous vaccine administration and booster vaccination alone were not met in our initial or sensitivity analysis, with mean concentrations of anti-S IgG being marginally lower in the simultaneous vaccination group. Viral neutralization assays against SARS-CoV-2 also suggested potential immune interference. The clinical impact of this effect is partially uncertain, as a threshold associated with minimal protection is not yet available for COVID-19 (i.e. antibody-based correlate of protection). However, it is conceivable that a lower level of specific antibodies might result in reduced protection against COVID-19. Especially in vulnerable populations, this could be deleterious. The antibody responses measured in mucosal lining fluid were comparable to those measured in blood, which could be explained by translocation of systemically induced antibodies to the mucosal surface. Although the induction of specific mucosal antibodies after an mRNA vaccine has been demonstrated before,^21^ this is the first time that mucosal antibody responses are measured after a COVID-19 booster vaccination.

Furthermore, no differences in influenza titers in serum were measured between groups, indicating that possible interference does not extend to the immune response against influenza.

The safety data obtained in the present study is in line with recent research that shows no clinically relevant increase in adverse events or side-effects after concurrent administration of a COVID-19 mRNA vaccine and an influenza vaccine compared to a COVID-19 vaccine alone.^22^ Although the primary outcome of that study was safety, and had been powered solely for that aim, immunogenicity was assessed as well. Concomitant vaccination of a second-dose of the primary series of COVID-19 vaccinations (not a post-primary series booster vaccination as assessed in the current study) with an influenza vaccine was presented as preserving binding antibody responses, which is not in accordance with the conclusions drawn from our study. Important discrepancies between both studies in addition to the vaccination stage (primary series versus booster) include the older age of TACTIC-participants (depending on trial arms, >10 years) and the methods used to assess immunogenicity. No mucosal antibodies or virus neutralization capacities are presented, the latter being a major influential factor in our study. Of note, the authors do not show antibody concentrations in their paper, making it difficult to assess the exact results. Another recent study that assessed the immunological interaction between another COVID-19 vaccine (mRNA-1273) and influenza vaccination did not identify any interference between a COVID-19 booster and an influenza vaccine, either.^23^ However, that study by Izikson et al. did not perform a formal statistical comparison between various vaccination schedules, nor was the neutralizing capacity of antibodies measured. The authors excluded (among others) the use of anticoagulants or previous vaccination by a viral vector vaccine, whereas the selection of participants for the TACTIC study did not include these criteria in order to have a more representative group of older adults. The additional selection criteria might have resulted in a study population with generally better responses to vaccination overall.

In addition to our study, an investigation into the immunological effects of NVX-CoV2373 COVID-19 vaccine and seasonal influenza vaccines did show a reduction in antibody responses against SARS-CoV-2 after concurrent vaccination,^24^ supporting our findings. The authors suggest that pre-existing immunological memory against the SARS-CoV-2 virus might minimize the possible interference; unfortunately, humoral immunological interference still cannot be ruled out in our booster-study. In general, the use of different vaccines in the various studies might have caused the differences in outcome.

An important aspect that remains to be studied in detail relates to the cellular and molecular mechanisms responsible for the effects observed. A possible explanation for the vaccine interference observed in our study may be the vaccination-induced type I interferons (IFNs) release, which may subsequently suppress the response to a simultaneously administered mRNA vaccine.^25^ However, this may be unlikely given the time that is needed to produce IFNs and the different vaccination sites used in this study (different arms). Impaired T cell function after simultaneous presentation of closely related variant epitopes has previously been described,^26^ but it remains to be demonstrated whether this mechanism might be responsible for the effects observed in the present study.

Our study also has limitations. One limitation of the TACTIC study design is the lack of epidemiological follow-up data, making it impossible to estimate vaccine effectiveness. Interpretation of the significance of serological results therefore remains an important area of research. Given that different virus variants seem to impair humoral immunity more than cellular responses,^17^ the lack of neutralizing antibodies might be compensated by T cell immunity which might be less affected by concurrent vaccine administration. Another limitation is the absence of data on T cells and memory B cells. To complement the findings from our study, future studies on T cell responses and memory B cells are warranted.

One important topic that has received little attention in vaccination studies is the long-term effect of vaccines on inflammation. Considering the known inflammatory side-effects of the novel COVID-19 vaccines, as well as rare (but sometimes severe) inflammatory complications in some vaccinated individuals,^27^^,^^28^ the assessment of long-term effects of the various vaccination schedules on the systemic inflammation is important. Although low systemic inflammation is associated with poorer vaccine responses,^29^ hyperinflammation can lead to more severe disease and prolonged upregulation of inflammatory markers is associated with increased cardiovascular risk.^30^ We found that, in line with previously mentioned work, the mRNA COVID-19 vaccine seems to increase several proteins associated with inflammation. The differentially upregulated proteins were not found in the subpopulation who subsequently received an influenza vaccine, arguing for an anti-inflammatory role for the influenza vaccine, in line with previous research.^11^ Potential long-term inflammatory effects of COVID-19 need to be considered and monitored in order to assess their relevance.

In conclusion, the TACTIC study cannot exclude the possibility of immune interference between an mRNA COVID-19 booster and an influenza vaccination when they are administered at the same time, resulting in a lower antibody concentration and reduced virus neutralizing activity against SARS-CoV-2. This is important to take into account when making public health decisions regarding vaccination schedules in populations at risk. More research is needed to understand the potential for immune interference, gain a broader understanding of the interaction between these vaccines and its clinical relevance, as well as long-term changes induced by these vaccines on the low-grade systemic inflammation.

## Contributors

All authors had full access to all the data in the study and had final responsibility for the decision to submit for publication. All authors contributed to the article and approved the submitted version.

EAD Study conceptualization/design, collected data, performed laboratory experiments, performed the analyses, wrote the first draft

BG Study design, performed laboratory experiments

EJMT Study design, performed the analyses

CGvK Performed laboratory experiments

HD Performed laboratory experiments

LvE Performed laboratory experiments

CvdGdJ Performed laboratory experiments

DvM Performed laboratory experiments

PCK Performed non-inferiority analyses

JD-A Study conceptualization/design

RvC Study conceptualization/design

JSvdM Study conceptualization/design, performed the analyses, wrote the first draft, supervised the work

MIdJ Study conceptualization/design

MGN Study conceptualization/design, supervised the work

## Data sharing statement

Pseudonymized participant data and samples will be stored for 15 years. If consent was given previously, these data can be shared with other infectious disease researchers after permission from the principal investigator.

## Declaration of interests

MGN is a scientific founder of TTxD, Lemba and BioTrip, and a member of the TTxD scientific advisory board. MGN has received research grants from TTxD and GSK. The other authors have no conflicts of interest.

## Appendix A Supplementary data

Supplementary Fig. S5

C1. Protocol_TACTIC.NL77590.091.21.v2.4.21102021_clean

Supplementary Methods S1

Caption for supplementary material
