# Supplementary material for: Timing and sequence of vaccination against COVID-19 and influenza (TACTIC): a single-blind, placebo-controlled randomized clinical trial
Source: Lancet Reg Health Eur. 2023 Apr 12;29:100628. doi: 10.1016/j.lanepe.2023.100628 (PMC10091277; doi:10.1016/j.lanepe.2023.100628)
Supplement: Supplementary Table S1 [file mmc1.docx]

| Primary analyses | Baseline GMC of anti-S IgG (BAU/ml) | GMC at day 21 (BAU/ml) | Estimate (95% CI) |
| --- | --- | --- | --- |
| ‘Combination’ vs Reference | 199∙0 (N=37) | 1683∙6 (N=37) | -0∙17910 (-**0∙3680** – 0∙009831) |
| ‘Influenza first’ vs Reference | 190∙4 (N=39) | 2347∙9 (N=39) | -0∙03462 (-0∙2211 - 0∙1518) |
| ‘COVID-19 Booster first’ vs Reference | 225∙1 (N=39) | 2136∙8 (N=38) | -0∙07555 (-0∙2632 – 0∙1121) |
| Reference ‘booster only’ | 258∙2 (N=38) | 2542∙8 (N=37) | *N/A* |
| Sensitivity analyses comparing ‘combination’ to ‘booster first (reference)’ | | | |
| 1. Adjusting for baseline anti-S IgG levels | | | -0∙1393 (**-0∙3018** – 0∙02328) |
| 2. + Adjusting for previous pneumococcal vaccination | | | -0∙1391 (**-0∙3034** – 0∙02510) |
| 3. + Adjusting for baseline anti-N IgG levels | | | -0∙1400 (**-0∙3023** – 0∙02235) |
| 4. + Excluding three participants with COVID-19 history | | | -0∙1435 **(-0∙3088** – 0∙02175) |
| 5. Combining ‘booster first’ with ‘booster only’ as one reference group | | | -0∙1165 (-0∙2507 - 0∙01767) |

*Supplementary table 1: non-inferiority analyses comparing anti-S IgG responses, each group compared to reference group ‘COVID-19 booster only’. If the lower limit of the 95% confidence interval lies above the non-inferiority margin of* ***-0∙3****, the result is considered non-inferior. All results are adjusted for multiple testing using a common reference group (Dunnett’s method).*

*GMC = geometric mean concentration; BAU = binding antibody units*
