# Supplementary material for: Timing and sequence of vaccination against COVID-19 and influenza (TACTIC): a single-blind, placebo-controlled randomized clinical trial
Source: Lancet Reg Health Eur. 2023 Apr 12;29:100628. doi: 10.1016/j.lanepe.2023.100628 (PMC10091277; doi:10.1016/j.lanepe.2023.100628)
Supplement: Supplementary Table S2 [file mmc2.docx]

| GMCs (BAU/ml) | Anti-S IgA | Anti-N IgA | Anti-RBD IgA | Anti-N IgG | Anti-RBD IgG |
| --- | --- | --- | --- | --- | --- |
| Influenza first  Baseline  Day 21 | 85∙1  511∙9 | 168∙9  141∙5 | 37∙1  116∙8 | 1∙5  1∙6 | 138∙7  1845∙1 |
| COVID-19 Booster first  Baseline  Day 21 | 92∙0  525∙9 | 115∙0  122∙6 | 38∙7  135∙5 | 1∙2  1∙5 | 155∙2  1466∙6 |
| Combination  Baseline  Day 21 | 99∙5  475∙9 | 146∙6  155∙3 | 33∙8  111∙2 | 1∙3  1∙4 | 118∙5  1129∙1 |
| COVID-19 Booster only  Baseline  Day 21 | 117∙0  856∙6 | 175∙3  173∙5 | 56∙6  211∙8 | 1∙7  2∙0 | 186∙6  1761∙0 |

*Supplementary table 2: geometric mean concentrations (GMCs) of antibodies at baseline and at 21 days after COVID-19 booster vaccination.*
