# Supplementary material for: Timing and sequence of vaccination against COVID-19 and influenza (TACTIC): a single-blind, placebo-controlled randomized clinical trial
Source: Lancet Reg Health Eur. 2023 Apr 12;29:100628. doi: 10.1016/j.lanepe.2023.100628 (PMC10091277; doi:10.1016/j.lanepe.2023.100628)
Supplement: Supplementary Table S3 [file mmc3.docx]

| Participants reporting side-effects within 14 days after first round of vaccination (no. of participants, %) | | | | | | | | | |
| --- | --- | --- | --- | --- | --- | --- | --- | --- | --- |
|  | **‘Influenza first’**  Influenza + placebo vacc (N=38) | | **‘COVID-19 Booster first’**  Booster + placebo vacc (N=38) | | **‘Combination’** Booster + Influenza vacc (N=38) | | **‘COVID-19 Booster only’**  Booster + placebo vacc (N=38) | | **p-value** |
| Fever | 0 | 0% | 2 | 5∙3% | 1 | 2∙6% | 3 | 7∙9% | 0∙311 |
| Redness at injection site | 3 | 7∙9% | 5 | 13∙2% | 5 | 13∙2% | 3 | 7∙9% | 0∙835 |
| Pain at injection site | 8 | 21∙1% | 24 | 63∙2% | 33 | 86∙8% | 27 | 71∙1% | **0∙000** |
| Swollen injection site | 1 | 2∙6% | 9 | 23∙7% | 3 | 7∙9% | 4 | 10∙5% | 0∙052 |
| Fatigue | 7 | 18∙4% | 8 | 21∙1% | 9 | 23∙7% | 6 | 15∙8% | 0∙896 |
| Myalgia | 3 | 7∙9% | 7 | 18∙4% | 13 | 34∙2% | 10 | 26∙3% | **0∙034** |
| Joint pain | 4 | 10∙5% | 3 | 7∙9% | 7 | 18∙4% | 3 | 7∙9% | 0∙470 |
| Headache | 8 | 21∙1% | 6 | 15∙8% | 12 | 31∙6% | 9 | 23∙7% | 0∙373 |
| Chills | 2 | 5∙3% | 4 | 10∙5% | 5 | 13∙2% | 5 | 13∙2% | 0∙623 |
| Nausea | 0 | 0% | 1 | 2∙6% | 0 | 0∙0% | 3 | 7∙9% | 0∙082 |
| Duration of side-effects (mean no. of days [range]) | 4∙2 [0-9] | | 4∙9 [0-7] | | 5∙3 [0-8] | | 5∙2 [0-11] | | **0∙033** |
| No. of participants experiencing severity score ≥ 4 | 3 | 7∙9% | 4 | 10∙5% | 2 | 5∙3% | 4 | 10∙5% | 0∙433 |
| Duration of severity score ≥ 4  (mean no. of days [range]) | 3∙3 (1-8) | | 1∙0 (1-1) | | 1∙0 (1-1) | | 1∙25 (1-2) | | 0∙418 |

*Supplementary table 3: occurrence of reported side-effects within 14 days after first round of vaccination. Results shown per study group.*
