# Supplementary material for: Timing and sequence of vaccination against COVID-19 and influenza (TACTIC): a single-blind, placebo-controlled randomized clinical trial
Source: Lancet Reg Health Eur. 2023 Apr 12;29:100628. doi: 10.1016/j.lanepe.2023.100628 (PMC10091277; doi:10.1016/j.lanepe.2023.100628)
Supplement: Supplementary Table S4 [file mmc4.docx]

| Side-effect | Relative Risk (95% CI) |
| --- | --- |
| Fever | 0∙629 (0∙339-1∙166) |
| Redness at injection site | 1∙333 (0∙518-3∙368) |
| Pain at injection site | 1∙429 (0∙884-2∙309) |
| Swollen injection site | 0∙836 (0∙420-1∙664) |
| Fatigue | 1∙271 (0∙651-2∙481) |
| Myalgia | 1∙173 (0∙685-2∙008) |
| Joint pain | 1∙719 (0∙648-4∙562) |
| Headache | 1∙189 (0∙679-2∙081) |
| Chills | 0∙969 (0∙496-1∙892) |
