# Supplementary figures and images for: Timing and sequence of vaccination against COVID-19 and influenza (TACTIC): a single-blind, placebo-controlled randomized clinical trial
Source: Lancet Reg Health Eur. 2023 Apr 12;29:100628. doi: 10.1016/j.lanepe.2023.100628 (PMC10091277; doi:10.1016/j.lanepe.2023.100628)

● Influenza first    ■ Booster first    ▲ Combination    ▼ Booster only

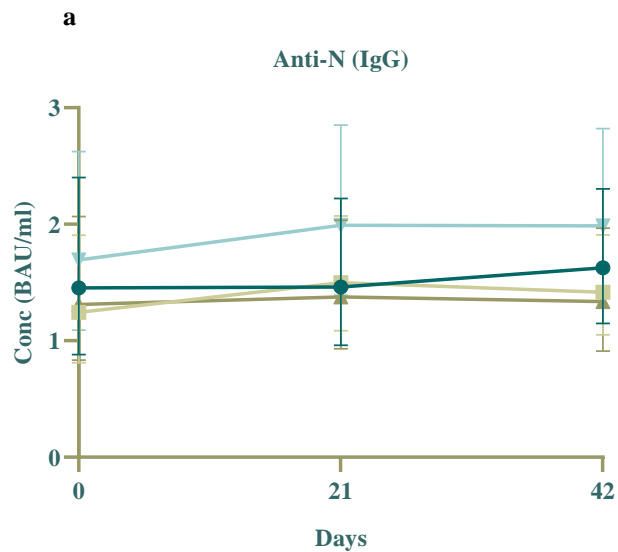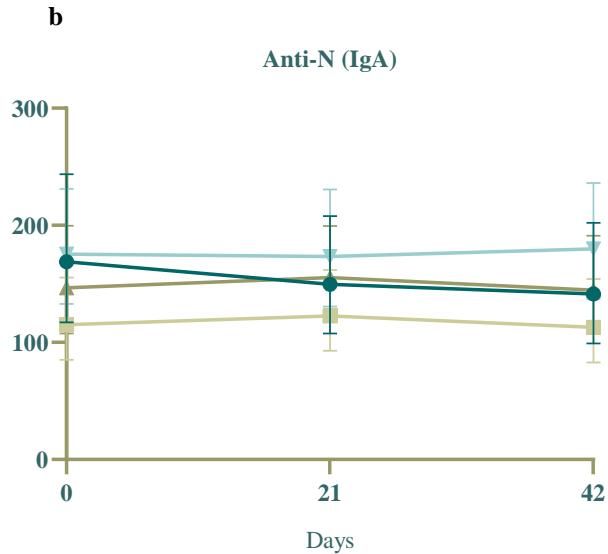

Supplement: Supplementary Fig. S2 [file mmc5.pdf]

● Influenza first    ■ Booster first    ▲ Combination    ▼ Booster only

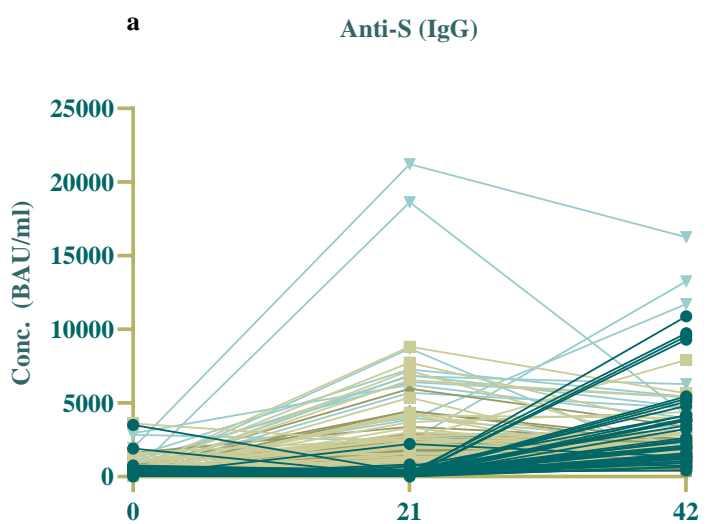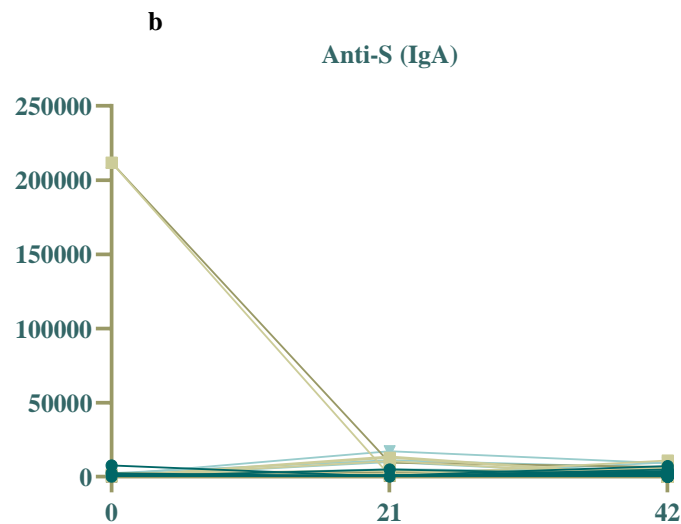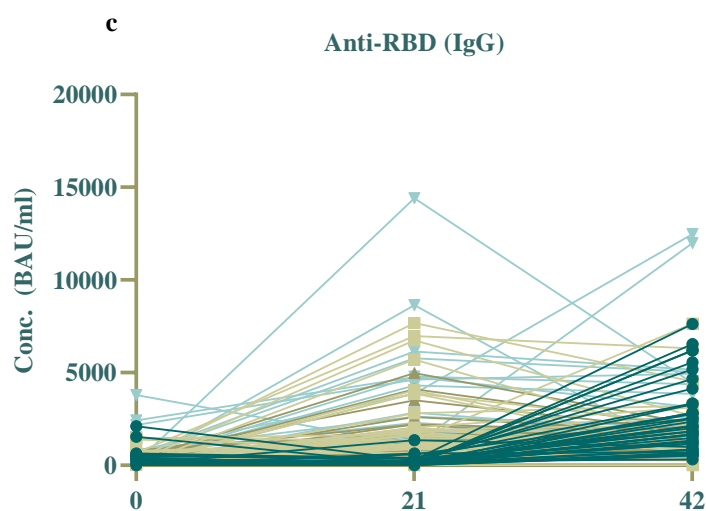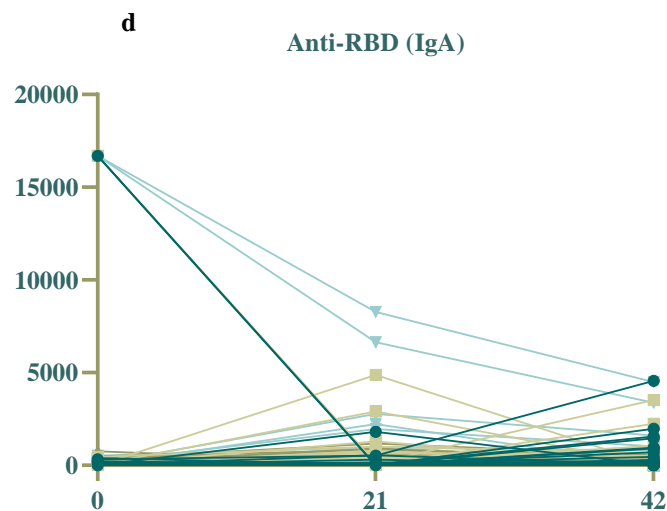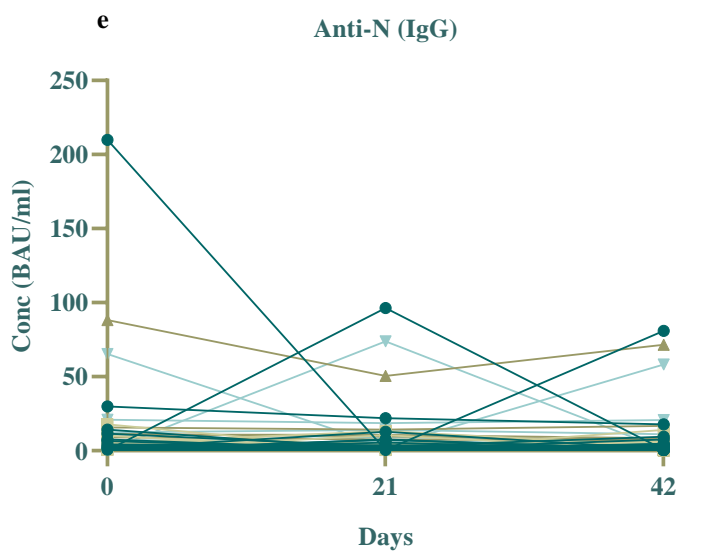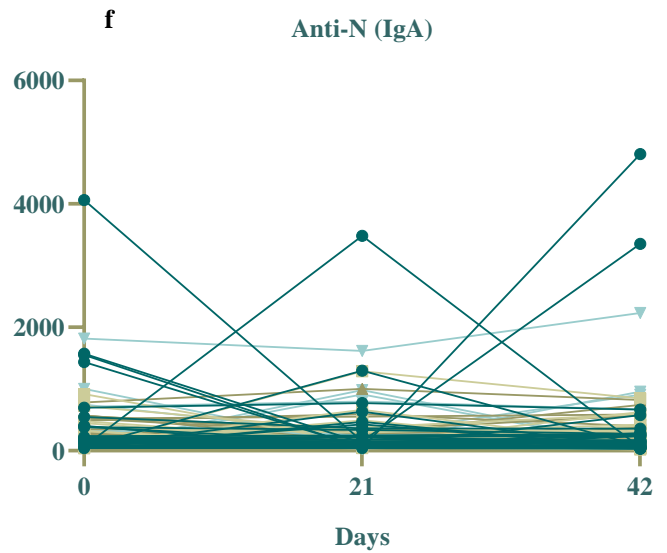

Supplement: Supplementary Fig. S3 [file mmc6.pdf]

● Influenza first    
 ■ Booster first    
 ▲ Combination    
 ▼ Booster only

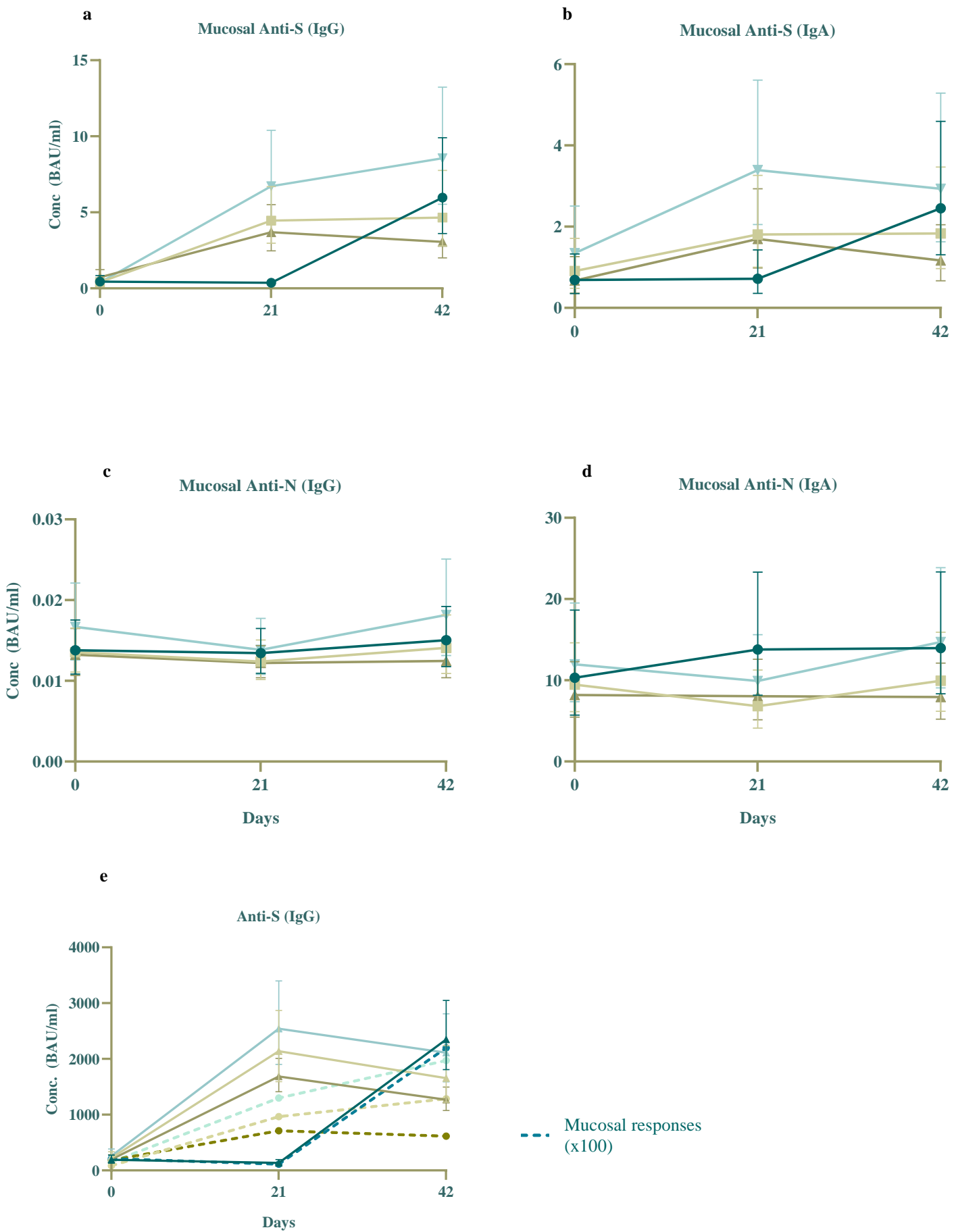

Supplement: Supplementary Fig. S4 [file mmc7.pdf]
