# Supplementary material for: Timing and sequence of vaccination against COVID-19 and influenza (TACTIC): a single-blind, placebo-controlled randomized clinical trial
Source: Lancet Reg Health Eur. 2023 Apr 12;29:100628. doi: 10.1016/j.lanepe.2023.100628 (PMC10091277; doi:10.1016/j.lanepe.2023.100628)
Supplement: Supplementary Fig. S5 [file mmc8.pdf]

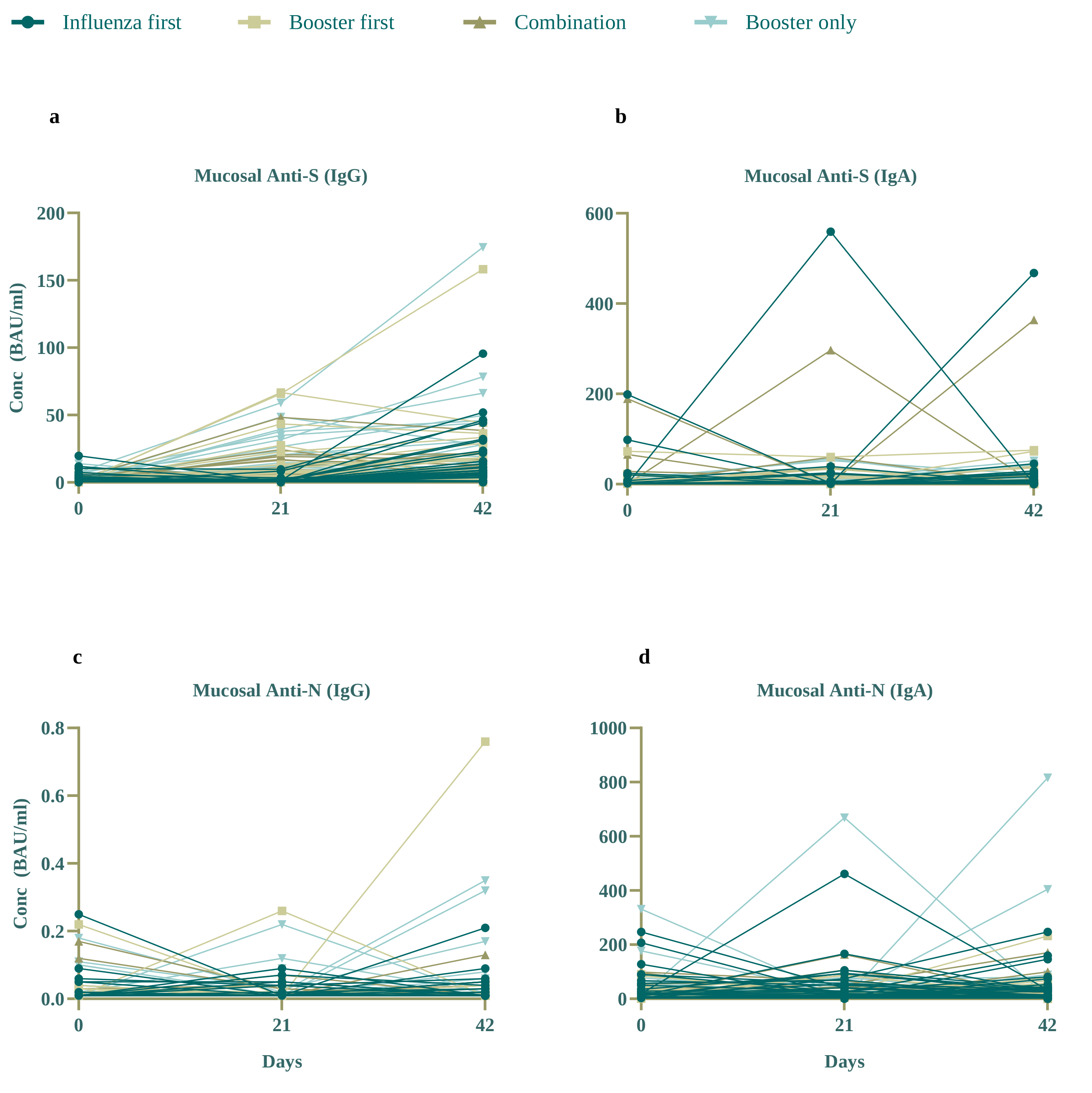

Influenza first
Booster first
Combination
Booster only
a
Mucosal Anti-S (IgG)
Conc (BAU/ml)
200
150
100
50
0
0
21
42
b
Mucosal Anti-S (IgA)
600
400
200
0
0
21
42
c
Mucosal Anti-N (IgG)
Conc (BAU/ml)
0.8
0.6
0.4
0.2
0.0
0
21
42
Days
d
Mucosal Anti-N (IgA)
1000
800
600
400
200
0
0
21
42
Days
