# Supplementary material for: Timing and sequence of vaccination against COVID-19 and influenza (TACTIC): a single-blind, placebo-controlled randomized clinical trial
Source: Lancet Reg Health Eur. 2023 Apr 12;29:100628. doi: 10.1016/j.lanepe.2023.100628 (PMC10091277; doi:10.1016/j.lanepe.2023.100628)
Supplement: C1. Protocol_TACTIC.NL77590.091.21.v2.4.21102021_clean [file mmc9.pdf]

TIMING AND SEQUENCE OF VACCINATION AGAINST COVID-19 AND INFLUENZA – A SINGLE BLIND, PLACEBO-CONTROLLED RANDOMIZED CLINICAL TRIAL (TACTIC)

Version 2.4

21-10-2021

PROTOCOL TITLE: 'TIMING AND SEQUENCE OF VACCINATION AGAINST COVID-19 AND INFLUENZA – A SINGLE BLIND, PLACEBO-CONTROLLED RANDOMIZED CLINICAL TRIAL'

**Protocol ID**	**TACTIC**
**Short title**	Vaccination strategy for COVID-19 and Influenza
**EudraCT number**	2021-002186-17
**Version**	2.3
**Date**	23-08-2021
**Coordinating investigator/project leader**	Elisabeth Dulfer, PhD candidate<br>Elisabeth.dulfer@radboudumc.nl<br>Radboudumc, Department of internal medicine, Radboud Centre of Infectious Diseases
**Principal investigator(s) (in Dutch: hoofdonderzoeker/ uitvoerder)**	Dr. Josephine van de Maat, postdoctoral researcher<br>Josephine.vandemaat@radboudumc.nl<br>Radboudumc, Department of internal medicine, Radboud Centre of Infectious Diseases
**Sponsor (in Dutch: verrichter/opdrachtgever)**	Radboudumc, Department of internal medicine, Radboud Centre of Infectious Diseases
**Subsidising party**	ZonMW
**Independent expert (s)**	Dr. Q. de Mast<br>Quirijn.demast@Radboudumc.nl<br>Radboudumc, department of internal medicine
**Laboratory**	*Laboratory of Internal Medicine, Radboudumc*<br>Dr. Jorge Dominguez Andres, postdoctoral researcher, Jorge.dominguezandres@Radboudumc.nl<br>Prof. Dr. Yang Li, system biologist<br>Dr. Cheng-Jian Xu, bioinformatician<br>*Laboratory of Medical Immunology, Radboudumc*<br>Dr. Marien de Jonge, associate professor, Marien.dejonge@Radboudumc.nl<br>*Department of Virology, Erasmus MC:*<br>Dr. Corine Geurts van Kessel, virologist
**Pharmacy**	Pharmay of Radboudumc<br>Contact person: dr. Rob ter Heine, pharmacist

PROTOCOL SIGNATURE SHEET

**Name**	**Signature**	**Date**
**Head of Department:**<br><br>Prof. Saskia Middeldorp		
**Principal Investigator:**<br><br>Dr. Josephine van de Maat		

TABLE OF CONTENTS

1. INTRODUCTION AND RATIONALE ... 8
2. OBJECTIVES ... 10
3. STUDY DESIGN ... 11
STUDY POPULATION ... 13
3.1 Population (base) ... 13
3.2 Inclusion criteria ... 13
3.3 Exclusion criteria ... 13
3.4 Sample size calculation ... 13
4. TREATMENT OF SUBJECTS ... 15
4.1 Investigational product/treatment ... 15
5. INVESTIGATIONAL PRODUCT ... 16
5.1 Name and description of investigational product(s) ... 16
5.2 Summary of findings from non-clinical studies ... 16
5.3 Summary of findings from clinical studies ... 16
5.4 Summary of known and potential risks and benefits ... 16
5.5 Description and justification of route of administration and dosage ... 17
5.6 Preparation and labelling of Investigational Medicinal Product ... 17
5.7 Drug accountability ... 17
6. METHODS ... 17
6.1 Study parameters/endpoints ... 17
6.1.1 Main study parameter/endpoint ... 17
6.1.2 Other study parameters ... 18
6.2 Randomisation, blinding and treatment allocation ... 19
6.3 Study procedures ... 19
6.4 Withdrawal of individual subjects ... 20
6.5 Replacement of individual subjects after withdrawal ... 21
6.6 Follow-up of subjects withdrawn from treatment ... 21
6.7 Premature termination of the study ... 21
7. SAFETY REPORTING ... 22
7.1 Temporary halt for reasons of subject safety ... 22
7.2 AEs, SAEs and SUSARs ... 22
7.2.1 Adverse events (AEs) ... 22
7.2.2 Serious adverse events (SAEs) ... 22
7.2.3 Suspected unexpected serious adverse reactions (SUSARs) ... 23
7.3 Annual safety report ... 23
7.4 Follow-up of adverse events ... 24
8. STATISTICAL ANALYSIS ... 25
8.1 Primary and secondary study parameter(s) ... 25
8.2 Other study parameters ... 25
8.3 Interim analysis ... 25
9. ETHICAL CONSIDERATIONS ... 26

9.1 Regulation statement 26
9.2 Recruitment and consent....26
9.3 Eligibility of minors or incapacitated subjects....26
9.4 Benefits and risks assessment, group relatedness....26
9.5 Compensation for injury 26
9.6 Incentives....27
10. ADMINISTRATIVE ASPECTS, MONITORING AND PUBLICATION 28
10.1 Handling and storage of data and documents 28
10.2 Monitoring and Quality Assurance....28
10.3 Amendments....29
10.4 Annual progress report....29
10.5 Temporary halt and (prematurely) end of study report....29
10.6 Public disclosure and publication policy....29
11. STRUCTURED RISK ANALYSIS....30
11.1 Potential issues of concern....30
11.2 Synthesis 31
12. REFERENCES 32

LIST OF ABBREVIATIONS AND RELEVANT DEFINITIONS

**ABR**	**General Assessment and Registration form (ABR form), the application form that is required for submission to the accredited Ethics Committee; in Dutch: Algemeen Beoordelings- en Registratieformulier (ABR-formulier)**
**AE**	**Adverse Event**
**AR**	**Adverse Reaction**
**CA**	**Competent Authority**
**CCMO**	**Central Committee on Research Involving Human Subjects; in Dutch: Centrale Commissie Mensgebonden Onderzoek**
**COVID**	**Corona virus disease**
**CV**	**Curriculum Vitae**
**DSMB**	**Data Safety Monitoring Board**
**EU**	**European Union**
**EudraCT**	**European drug regulatory affairs Clinical Trials**
**GCP**	**Good Clinical Practice**
**GDPR**	**General Data Protection Regulation; in Dutch: Algemene Verordening Gegevensbescherming (AVG)**
**IB**	**Investigator's Brochure**
**IC**	**Informed Consent**
**IMP**	**Investigational Medicinal Product**
**IMPD**	**Investigational Medicinal Product Dossier**
**METC**	**Medical research ethics committee (MREC); in Dutch: medisch-ethische toetsingscommissie (METC)**
**(S)AE**	**(Serious) Adverse Event**
**SPC**	**Summary of Product Characteristics; in Dutch: officiële productinformatie IB1-tekst**
**Sponsor**	**The sponsor is the party that commissions the organisation or performance of the research, for example a pharmaceutical company, academic hospital, scientific organisation or investigator. A party that provides funding for a study but does not commission it is not regarded as the sponsor, but referred to as a subsidising party.**
**SUSAR**	**Suspected Unexpected Serious Adverse Reaction**
**UAVG**	**Dutch Act on Implementation of the General Data Protection Regulation; in Dutch: Uitvoeringswet AVG**
**WMO**	**Medical Research Involving Human Subjects Act; in Dutch: Wet Medisch-wetenschappelijk Onderzoek met Mensen**

SUMMARY

Rationale: Vaccination is considered the way out of the current COVID-19 pandemic, with detrimental effects on morbidity, mortality and societal concerns. The general assumption is that booster shots (re-vaccination with the same or variant vaccines) will be needed this autumn to ensure ongoing protection against SARS-CoV-2; coinciding with annual influenza vaccination campaigns, thus posing a tremendous organisational and scientific challenge. The specific combination in which both vaccines can be administered to produce optimal protection for both viruses is not examined yet.

Objectives:

The **overall aims** of this study are (1) to evaluate immunogenicity and safety of combined influenza- and SARS-CoV-2 vaccinations, and (2) to understand underlying immunological mechanisms of potential interference. .

Study design: Single-blind, placebo-controlled randomized clinical trial

Study population: 140 human volunteers aged >60 years, with SARS-CoV-2-vaccination in the past 4-12 months, and no acute illness or immune suppression.

Intervention: participants will be randomized between four treatment arms (n=35/arm):

1. Influenza + placebo vaccine followed by booster Comirnaty vaccine;
2. Booster Comirnaty + placebo vaccine followed by influenza vaccine;
3. Influenza vaccine + booster Comirnaty vaccine, followed by placebo vaccine;
4. Comirnaty booster + placebo vaccine, followed by placebo vaccine (reference group);

Main study parameters/endpoints: Primary endpoints are geometric mean titers of IgG against S-protein in serum at 21 days after last vaccination. Secondary endpoints are seroconversion of IgG to the SARS-CoV-2 spike protein at day 21 after the COVID-19 booster vaccines; virus neutralization assays for the standard SARS-CoV-2 variant, as well as for the B1.1.7, B.1.617.2 and B1.351 variants; IgA and IgG responses against RBD- and S- and N-protein in MLF and serum at baseline, 21 days after each vaccination; IgG and IgA against influenza antigens in mucosal lining fluid (MLF) and serum at baseline and 211 days after each vaccination; specific anti-SARS-CoV-2 T-cell responses against standard SARS-CoV-2 variant, as well as for the B1.1.7, B.1.617.2 and B1.351 variants; local reactions at injection site or systemic reactions after vaccination; serious adverse events and other adverse events. Exploratory endpoints are myeloid cells transcriptional/epigenetic program and function; assessment of systemic inflammation by targeted proteome analysis and changes in circulating metabolome.

Nature and extent of the burden and risks associated with participation, benefit and group relatedness: Based on previous experience in published and ongoing randomized controlled trials, the risks of Influenza vaccination and SARS-CoV-2 vaccination are considered negligible. Participants will visit the study clinic 3 times over the course of 42 days for vaccination, venous blood + MLF samples. Venepuncture imposes negligible risk to participants. Collection of MLF is non-invasive and poses no risk to participants. A benefit for participants of the trial is the opportunity to receive a COVID-19 vaccine booster before the (possible) implementation in a routine campaign and the opportunity to receive an influenza vaccine ahead of the routine national vaccination campaign.

1. INTRODUCTION AND RATIONALE

After the first official case of coronavirus disease 2019 (COVID-19) in China in December 2019, the SARS-CoV-2 virus that causes the infection quickly spread around the world, with the first European disease case reported merely one month later. As of May 2021, there have been over 160 million confirmed cases of COVID-19 globally, with a number of deaths caused by the disease approaching 3.5 million (1). After the authorities in US, UK and EU issued emergency use authorizations of COVID-19 vaccines at the end of 2020 and beginning of 2021, the situation slowly started to improve. Although most countries currently vaccinating their inhabitants have experienced logistic difficulties and delayed deliveries of vaccines, the number of vaccinated individuals is on the rise and it has become clear that vaccinating is an effective strategy to decrease morbidity and mortality. In March, the Dutch RIVM (National Institute for Public Health and the Environment) reported a clear reduction in infection rates and hospital admissions in persons aged 80+ years, eight weeks after the first vaccinations in that group (2). Four different vaccines are currently administered in the Netherlands, produced by Pfizer/BioNTech (Comirnaty; BNT162b2), Moderna (mRNA-1237), Janssen (Ad26.COV2.s) and AstraZeneca (ChAdOx1-S), although it is expected that in the future especially Cominarty will be used. The longevity of the protection provided by these vaccines has yet to be determined and clinical trials doing so are currently running. Recently published research indicates that mRNA-1237 vaccination could provide durable humoral immunity (3), but antibody responses after COVID-19 have been shown to vary greatly amongst individuals (4) and cellular responses have not yet been well defined. In addition, several new SARS-CoV-2 variants have emerged in various countries, and there are indications that some of them have the capacity to at least partially evade the protection provided by vaccination (5). Due to these observations, the general assumption is that booster shots (with the same or variant vaccines) for re-vaccination may be needed this autumn to avoid a winter surge: it is also possible that annual COVID-19 vaccinations may be needed, similar to the vaccination against influenza.

One existing annual vaccination programme is the immunization against influenza in autumn of risk groups, like the elderly. In the Netherlands, each year more than 3 million persons receive their flu shot. Presuming that COVID-19 boosters are needed around the same time, this poses a tremendous organisational and scientific challenge: the specific order in which the two vaccines should be combined to produce optimal protection for both viruses is not known yet. Co-administration would provide many logistic advantages, but the efficacy of concurrent vaccination of these particular vaccines has not been assessed. The safety of this co-administration is currently under investigation in a trial in the UK (ComFluCOV study, https://comflucov.blogs.bristol.ac.uk/); we know from personal correspondence with the UK investigators that no safety signal has occurred during this trial, although official analyses have not been released yet.

Vaccine interference is a known phenomenon, but little studied in the context of the COVID-19 pandemic. The immunological and clinical interaction when COVID-19 vaccines are combined with the influenza vaccine is not known and could theoretically result in both positive and negative responses, ranging from enhanced immunity against both viruses to inhibition of immune responses to one or both of the viruses. It may also be possible that a specific order in which the vaccines are given could enhance or inhibit the efficacy of the vaccinations. Research has been done in the past concerning the co-administration of different vaccines, e.g. the seasonal influenza vaccine concurrent with the pandemic H1N1 vaccine (6), the combination of a live-attenuated influenza vaccine in young children with other commonly administered childhood vaccinations (7, 8), or combinations between vaccination programs in developing countries containing live (BCG, measles) and inactivated (e.g. DTP) vaccines. While some of those studies showed no clinically relevant differences in immune responses when influenza vaccine was administered simultaneously or apart with other vaccines, other studies have shown strong interference between measles, BCG and DTP vaccinations (9, 10). Another previous study suggested that vaccination with an inactivated influenza vaccine can

alter the immune response to COVID-19 (Debisarun et al, in preparation). Nothing is known regarding the impact of mRNA vaccines () with regard to vaccine interference, and it is difficult to hypothesise about plausible outcomes regarding efficacy and safety. The first crucial question is to know whether influenza vaccination prior to, after, or combined with COVID-19 vaccination will not inhibit the immune response against SARS-CoV-2 induced by the novel specific vaccines. Investigating this is of profound importance for the vaccination strategy in the coming years.

We aim to shed light on this important scientific and public health problem by investigating the effects of different sequences of the influenza vaccination and the COVID-19 vaccination most likely to be used long-term in the Netherlands on the immune responses and side-effects in elderly. Our objectives are thus two-fold: from a public health perspective, we want to establish the optimal vaccination strategy for COVID-19 and influenza vaccination; from a scientific perspective, we aim to understand the potential immune interference between these vaccines in terms of immunogenicity and safety.

2. OBJECTIVES

The **overall aim** of this study is to evaluate immunogenicity and safety of combined influenza- and SARS-CoV-2 vaccinations, and to understand underlying immunological mechanisms of potential interference.

Primary Objective:
To study the impact of different sequences of combined influenza- and SARS-CoV-2 vaccination in elderly on IgG responses against S-protein in serum at 21 days after last vaccination.

Secondary Objectives:
To study the impact of different sequences of combined influenza- and SARS-CoV-2 vaccination in elderly on:

- Seroconversion of IgG to the SARS-CoV-2 spike protein at day 21 after the COVID-19 booster vaccines. Seroconversion of antibodies is defined as a change from seronegative at baseline (pre- COVID-19 booster vaccine) to seropositive or a ≥four-fold titer increase if the participant is seropositive at baseline
- Virus neutralization assays for the standard SARS-CoV-2 variant, as well as for the B1.1.7, B.1.617.2 and B1.351 variants
- IgA and IgG responses against RBD- and S- and N-protein in MLF and serum at baseline, 21 days after each vaccination
- IgG and IgA against influenza antigens in MLF and serum at baseline, 21 days after each vaccination.
- Specific anti-SARS-CoV-2 T-cell responses against standard SARS-CoV-2 variant, as well as for the B1.1.7, B.1.617.2 and B1.351 variants
- Local reactions at injection site or systemic reactions after vaccination
- Serious adverse events and other adverse events.

Exploratory endpoints:

- Myeloid cells transcriptional/epigenetic program and function
- Innate immune responses as assessed by proinflammatory production by myeloid immune cells
- Assessment of systemic inflammation by targeted proteome analysis
- Changes in circulating metabolome

3. STUDY DESIGN

A single-blind, placebo-controlled, single-center randomized clinical trial will be performed among volunteers who are ≥60 years of age, with prior vaccination against SARS-CoV-2 in the past 4-12 months and without acute illness or immune suppression.. Participants will be blinded to the intervention, to ensure unbiased reporting of side effects after vaccination. Participants will be recruited by open invitation. Those interested to participate will receive written information on the study. Participants will be screened for eligibility by means of a phone call and those eligible will be invited for the first study visit. After informed consent has been given, baseline information will be recorded and participants will be randomized to one of four treatment arms (n=35 volunteers/arm, stratified by sex):

1. Influenza + placebo vaccine followed by booster Comirnaty vaccine;
2. Booster Comirnaty + placebo vaccine followed by influenza vaccine;
3. Influenza vaccine + booster Comirnaty vaccine, followed by placebo vaccine;
4. Comirnaty booster + placebo vaccine, followed by placebo vaccine (reference group);

Choice of the COVID-19 vaccines to be investigated. There are currently four different COVID-19 vaccines approved in the EU. The use of ChAdOx1-S (by Astra-Zeneca) and Ad26.COV2.S (by Janssen) have rare but severe thromboembolic complications. Their use in the Netherlands is currently restricted, the contract with Astra-Zeneca was not extended beyond this year and with Janssen will also likely end. The other two vaccines are currently mainly used in The Netherlands and are expected to be continued, with Cominarty being preferentially used. Therefore, we will focus the study on the interaction between Comirnaty (by Pfizer-BioNTech) with the influenza vaccine.

See Figure 1 for the graphic design of the trial and Table 1 the vaccination and sample collection schedule. At each study visit blood and mucosal samples will be collected before vaccination. On day 42 at the end of the study, all volunteers who received a placebo instead of influenza vaccination will be offered to be vaccinated against influenza.

Table 1. Vaccination and sample collection schedule

Group	N	Day 1	Day 21	Day 42
1	35	**Influenza** + placebo	sample collection + **Comirnaty** booster	sample collection
2	35	**Comirnaty** booster + placebo	sample collection + **Influenza**	sample collection
3	35	**Influenza** + **Comirnaty** booster	sample collection + **Placebo**	sample collection
4 (ref)	35	**Comirnaty** booster + placebo	sample collection + **Placebo**	sample collection + **Influenza**

Figure 1. Graphic design of the TACTIC study. All steps will be documented.

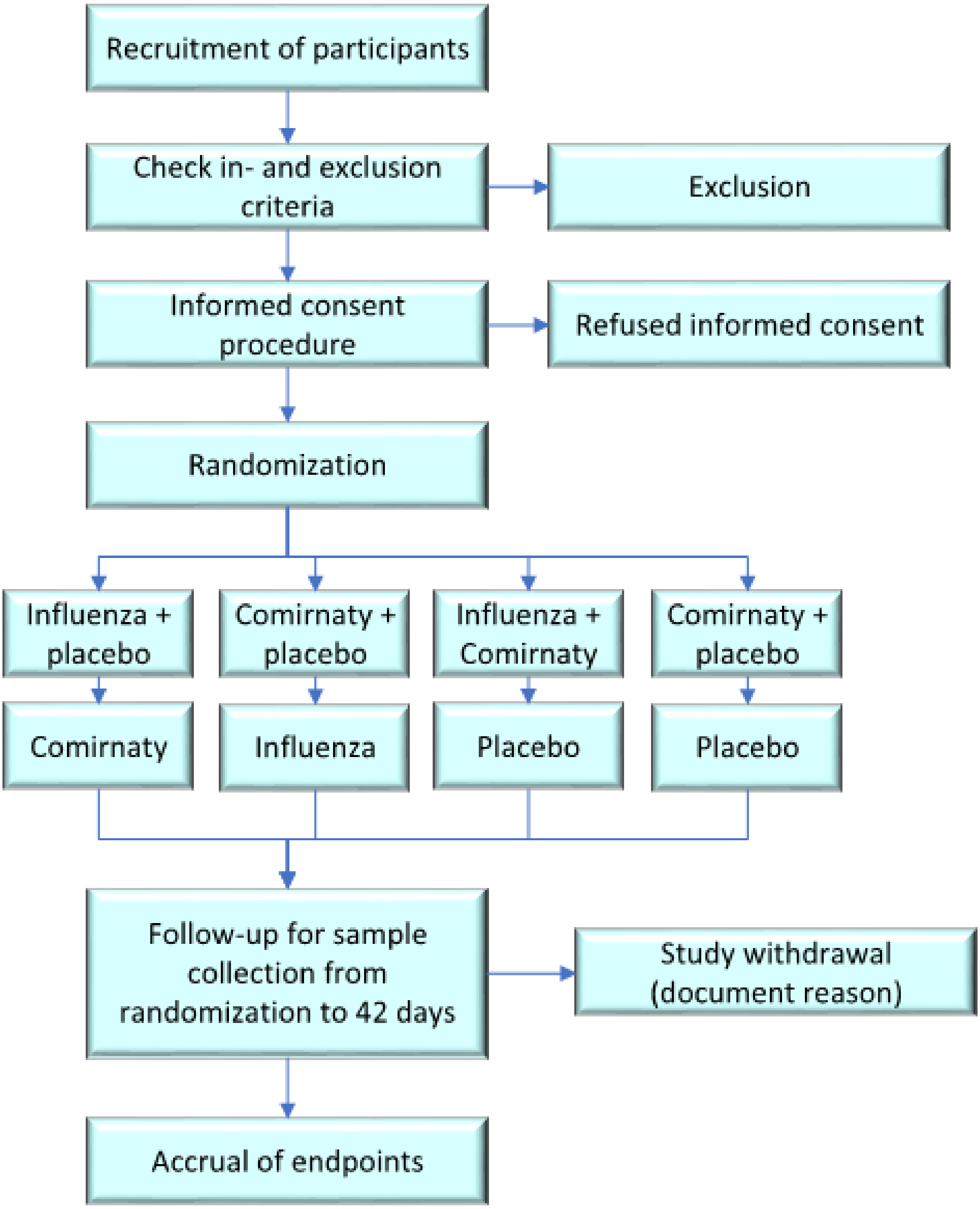

STUDY POPULATION

3.1 Population (base)

The research population will consist of people aged 60 and above, who are currently the main target population for the yearly influenza vaccination and also likely the group to receive future COVID-19 vaccine boosters. Recruitment of participants is planned from September to November 2021 and will be done by open invitation using advertisements in local newspapers. In addition, we will invite elderly participants of previous clinical trials of our department, who gave consent to be approached for future studies. We expect to recruit sufficient participants in this manner.

3.2 Inclusion criteria

In order to be eligible to participate in this study, a subject must meet all of the following criteria:

- Age ≥60 years
- Received a COVID-19 vaccine 4-12 months prior to enrolment

3.3 Exclusion criteria

A potential subject who meets any of the following criteria will be excluded from participation in this study:

- Vaccinated with any SARS-CoV-2 vaccine, or history of documented COVID-19, within four months prior to enrolment
- Vaccination in past 6 months with influenza vaccine
- Vaccination other than COVID-19 or Influenza within 6 months prior to study, or expected during study period – with the exception of the routine vaccination campaign against *Pneumococcus*, or vaccines administered in the context of this study
- Immunocompromised (either by co-morbidities or induced by medication)
- Acute illness past 2 weeks
- Known allergy or history of anaphylaxis or other serious adverse reactions to vaccines
- Participation in another drug trial
- Legally incapacitated or unwilling to provide informed consent

3.4 Sample size calculation

The required sample size to evaluate non-inferiority of the primary endpoint has been calculated based on geometric mean IgG titers after vaccination with the COVID-19 vaccine (from Pfizer) alone (11-15). Table 3 shows the required sample size for various non-inferiority margins and power limits, based on the following assumptions: estimated standard deviation of 0.38, non-inferiority under the assumption of no difference in IgG titers (delta=0), with various non-inferiority margins (-0.2 to -0.4 on log10 scale), one-sided non-inferiority alpha of 2.5%, power of 90% or 80%.

Non-inferiority margin on log10 base	Required number of participants per group at 80% power	Required number of participants per group at 90% power
-0.2	56	77
-0.3	25	**35**
-0.4	15	20

Table 3. Sample size calculation primary endpoint

We will include 35 participants per intervention group, which will provide 90% power to evaluate non-inferiority of ‘prior influenza vaccination’, ‘later influenza vaccination’ and ‘concomitant influenza/COVID-19 vaccination’, compared to ‘COVID-19 vaccination alone’, considering -0.3 non-inferior. It must be noted that this is a conservative calculation, since we worked with the available evidence on IgG titers after primary vaccination, while the expected IgG titer variability (SD) after booster vaccination is likely to be much lower, but currently no evidence is available on this.

4. TREATMENT OF SUBJECTS

4.1 Investigational product/treatment

Participants in study groups 1-3 will be vaccinated with the licensed Influenza vaccine of the season (for 2021/2022 either Vaxigrip Tetra (Sanofi Pasteur Europe) or Influvac Tetra (Abbott bv). All participants will receive the same type of influenza vaccine).
Participants in all groups 1-4 will be vaccinated with the licensed COVID-19 mRNA vaccine by Pfizer/BioNTech (Comirnaty; BNT162b2).

Placebo vaccine: intramuscular injection of sterile 0.9% NaCl.

All study vaccines will be administered using the standard vaccination techniques for these vaccines: intramuscular injection in the deltoid muscle in the upper arm.

5. INVESTIGATIONAL PRODUCT

5.1 Name and description of investigational product(s)

Influenza vaccine is an inactivated vaccine, containing surface antigens of Influenza A and B. it is indicated in The Netherlands for annual vaccination before the winter surge, to prevent influenza in risk groups (mainly elderly and people with underlying chronic diseases). Each year, a new vaccine is developed to provide optimal protection against the circulating influenza strains. For the current season both Vaxigrip Tetra and Influvac Tetra are used for the routine vaccination campaign in adults.
Comirnaty (BNT162b2, COVID-19 vaccine by Pfizer/BioNTech) is the first COVID-19 vaccine that was licensed in the European Union, approved on 21 December 2020 for emergency use by the European Medicine Agency (EMA), and is currently used in all European countries.

5.2 Summary of findings from non-clinical studies

Due to the medical emergency of COVID-19, which emerged as disease only little more than one year ago, and the urgent need of vaccines against SARS-CoV-2, there are no available non-clinical studies assessing the potential vaccine interference between mRNA COVID-19 vaccines and influenza vaccine.

5.3 Summary of findings from clinical studies

Pfizer/BioNTech COVID-19 vaccine have been shown to be highly effective against COVID-19. On immunological level, Pfizer/BioNTech COVID-19 vaccine was safe and has been demonstrated to induce strong neutralizing antibodies(12). Clinical efficacy upon introduction in the population in real-life setting reached 92% (16). For a summary of findings from clinical studies we also refer to the Summary of Product Characteristics (SPC), pages 4-8. No data are currently available regarding the potential vaccine interference between COVID-19 vaccines and influenza vaccine.

5.4 Summary of known and potential risks and benefits

For details of known and potential risks and befits we refer to the Summary of Product Characteristics (SPC), pages 3-6.
In summary, the most frequent adverse reactions in participants of the **Pfizer** clinical trial were injection site pain (> 80%), fatigue (> 60%), headache (> 50%), myalgia and chills (> 30%), arthralgia (> 20%), pyrexia and injection site swelling (> 10%) and were usually mild or moderate in intensity and resolved within a few days after vaccination. Benefit: the efficacy of the Pfizer vaccine for symptomatic infections was 94.6% (95% credible interval of 89.9% to 97.3%). The beneficial effect of an added booster vaccine is currently not known.

The safety of **Vaxigrip** Tetra was assessed in six clinical trials, including 1392 participants aged >60 years. Most reactions occurred within the first 3 days following vaccination and resolved spontaneously within 1 to 3 days after onset. The intensity of these reactions was generally mild. The most frequently reported adverse reactions after vaccination observed in the clinical studies for Vaxigrip Tetra were vaccination site pain (54%), headache (15.6%) and myalgia (13.9%).

The safety of **Influvac** sub-unit Tetra was assessed in two clinical trials in which healthy adults 18 years of age and older. Most reactions occurred within the first 3 days following vaccination and resolved spontaneously within 1 to 3 days after onset. The intensity of these reactions was generally mild. The most frequently reported adverse reactions after vaccination observed in the clinical studies for Influvac sub-unit Tetra were vaccination site pain, fatigue and headache (all 'very common', >10%); 'common' adverse reactions (>1%) were myalgia, arthralgia and shivering.

5.5 Description and justification of route of administration and dosage

According to standard vaccination practices, Comirnaty is administered intramuscularly after dilution as a course of 2 doses (0.3 mL each) at least 21 days apart; in our study, we will use a single-dose booster. Influenza vaccination is administered intramuscularly in one 0.5 mL dose. Placebo will be administered as a dose of 0.5ml NaCl 0.9%.
All vaccines will be administered in the deltoid muscle of the upper arm. Route of administration follows the recommendation of the manufacturers.

5.6 Preparation and labelling of Investigational Medicinal Product

The influenza vaccines used in this study are investigational medicinal products and will be dispensed by the department Pharmacy of the Radboudumc. In accordance with item 57 of the Regulation (EU) on clinical trials on medicinal products for human use (No 536/2014), which has been adopted by the Radboudumc and the ethical review board Arnhem-Nijmegen, no additional labeling labelling according to GMP annex 13 is required in this study, because an authorized medicinal product is used in this clinical trial and blinding is guaranteed procedurally, as participants will not see what will be administered (please refer to the new clinical trial regulation found at https://ec.europa.eu/health/sites/health/files/files/eudralex/vol-1/reg_2014_536/reg_2014_536_en.pdf).

5.7 Drug accountability

In line with the GCP, the investigator is responsible for maintaining drug accountability logs. Parts of this process may be delegated to other persons like a pharmacist and this will be noted in delegation logs.

6. METHODS

6.1 Study parameters/endpoints

6.1.1 Main study parameter/endpoint

The primary study end point will be:

- Geometric mean titers of IgG against S-protein in serum at 21 days after last vaccination.

Secondary study end points will be:

- Seroconversion of IgG to the SARS-CoV-2 spike protein at day 21 after the COVID-19 booster vaccines. Seroconversion of antibodies is defined as a change

from seronegative at baseline (pre- COVID-19 booster vaccine) to seropositive or a ≥four-fold titer increase if the participant is seropositive at baseline
- Virus neutralization assays for the standard SARS-CoV-2 variant, as well as for the B1.1.7, B.1.617.2 and B1.351 variants
- IgA and IgG responses against RBD- and S- and N-protein in MLF and serum at baseline, 21 days after each vaccination
- IgG and IgA against influenza antigens in MLF and serum at baseline, 21 days after each vaccination
- Specific anti-SARS-CoV-2 T-cell responses against standard SARS-CoV-2 variant, as well as for the B1.1.7, B.1.617.2 and B1.351 variants
- Local reactions at injection site or systemic reactions after vaccination
- Serious adverse events and other adverse events.

Exploratory endpoints:
- Myeloid cells transcriptional/epigenetic program and function
- Assessment of systemic inflammation by targeted proteome analysis
- Changes in circulating metabolome
- Innate immune responses as assessed by proinflammatory production by myeloid cells

6.1.2 Other study parameters

The following variables will be collected at baseline (table 2).

Table 2: Baseline variables collected for the trial

Variable
Date of inclusion
Sex
Age at inclusion
Height and weight (BMI)
Smoking history
Influenza vaccine received during preceding season
Timing and type of previous COVID-19 vaccination
History of BCG vaccination

The following personal data will be collected at baseline (Table 3). These data will be saved separately from the other study data.

Table 3: Personal data collected for the trial

Variable
Participant name
Participant date of birth
Participant phone number
Participant e-mail address
Participant postal address

At the end of follow-up the following data will be collected (table 4):

Table 4: Data collected during the study (not related to study endpoints)

Variable
Pneumococcal vaccination during study period (yes/no; vaccination date)
Any other vaccination (except study vaccines) during study follow-up (yes/no; name, vaccination date)

6.2 Randomisation, blinding and treatment allocation

Participants will be randomized in four treatment arms as previously described (see section 3. Study design). Randomization will be stratified by sex (male:female) and done by computer, using Castor Electronic Data Capture (Castor EDC). Allocation to treatment arms will be blinded to participants only.

6.3 Study procedures

<u>Before participation:</u>

- Participants will be informed about the study by open invitation through advertisements in local newspapers or by invitation based on previously given consent in other clinical trials of the department.
- Participants that are interested will receive written information about the study (including informed consent form) and invited for an telephone appointment.
- During a telephone appointment eligibility criteria will be checked, oral information will be given and questions from participants will be answered. The participant will be offered at least 24 hours to consider participation, after which they will sign the informed consent and send to the study staff.

<u>For participants:</u>

- After receiving the signed informed consent, randomization will be performed in Castor EDC, and participants will be scheduled for a first Study Visit with study staff.
- Study Visit 1:
  - Baseline information will be collected in eCRF in Castor.
  - Participants will receive information about their upcoming appointments.
  - (Before vaccination): Venous blood sample and nasal sample (MLF) is taken from all participants.
  - Participants receive ‘study vaccine 1’ by a study team member (including possible placebo)
  - After vaccination, participants will be observed for 15 minutes.
- Study Visit 2 (Day 21):
  - Baseline information will be collected in eCRF in Castor
  - (Before vaccination) Venous blood sample & MLF
  - Administration ‘Study vaccine 2’ by a study team member (possible placebo)
  - After vaccination, participants will be observed for 15 minutes.
- Study Visit 3 (Day 42):
  - Venous blood sample & MLF
  - Participants who did not receive an influenza vaccination will be offered to receive influenza vaccine

MLF will be collected by study staff by the use of the Nasosorption™ FXi nasal sampling device (Hunt Developments, UK). A synthetic absorptive matrix (SAM) strip will be gently inserted into the nostril of the participant and placed along the surface of the inferior turbinate to allow MLF absorption for 60 seconds, after which the SAM strip will be placed back in the protective plastic tube and stored at -20 until further processing (17). The procedure may tickle slightly but is painless.

- Starting both ‘vaccine visits’ until 14 days after vaccination: participants will keep a symptom diary to record side effects/adverse events. This will be discussed with participants before they receive each of their successive vaccinations. As will be indicated in the diary and oral/written information participants receive, recording of side effects / adverse events after each vaccination can stop after experiencing 3 successive days without any symptoms.
- At each visit, patients will fill out a short questionnaire assessing their possible encounters with SARS-CoV-2 testing and related hospital admissions.
- Day 1 until end of study (continuous): SAE recording.
- Participants are reminded about the coronavirus restrictions as imposed by the government and urged to strictly adhere to them.

Table 5. Schedule of events.

**Visit #**	Screening and randomization		**1**	**2**	**3**	**exit**
**visit timing**			vac 1	vac 2	vac 2 + 21 days	
**target visit day (window)**	**-30 to 1**		**1**	**21 (+/-2)**	**42 (+/-3)**	
**visit type**	Screening and randomization		vaccine 1	vaccine 2	sample	exit
**written informed consent**	x					
**inclusion/exclusion criteria**	x					
**demographics**	x					
**medical history / medication use**	x					
**Randomization**		x				
**venous blood sample (50mL)**			x	x	x	
**nasal sample (MLF)**			x	x	x	
**vaccination**			x	x		
**post-vaccination observation**			x	x		
**solicited AE recording**			x	x		
**unsolicited AE recording**			x	x		
**SAE recording**			x	x	x	x
**end-of-study questionnaire**						x

6.4 Withdrawal of individual subjects

Subjects can leave the study at any time for any reason if they wish to do so without any consequences and without the need for explanation. The investigator can decide to withdraw a subject from the study for urgent medical reasons.
Subjects who test positive for COVID-19 during the study, will be excluded from further analysis as both immunological and safety data will not be comparable anymore. These subjects will however be offered an influenza vaccine at their end of study, if they had not received it yet as part a study vaccination.

6.5 Replacement of individual subjects after withdrawal

Participants will only be replaced in case of withdrawal before the administration of the first vaccination. This will be limited to 10%.

6.6 Follow-up of subjects withdrawn from treatment

When participants want to leave the study or are withdrawn by the investigator, efforts will be made to ensure follow-up for safety endpoints.

6.7 Premature termination of the study

Based on the collected safety data (including severity of symptoms and adverse events), decisions can be made for the study as a whole, or for a certain treatment arm, to be terminated before the scheduled end.

7. SAFETY REPORTING

7.1 Temporary halt for reasons of subject safety

In accordance to section 10, subsection 4, of the WMO, the sponsor will suspend the study if there is sufficient ground that continuation of the study will jeopardise subject health or safety. The sponsor will notify the accredited METC without undue delay of a temporary halt including the reason for such an action. The study will be suspended pending a further positive decision by the accredited METC. The investigator will take care that all subjects are kept informed.

7.2 AEs, SAEs and SUSARs

7.2.1 Adverse events (AEs)

Adverse events are defined as any undesirable experience occurring to a subject during the study, whether or not considered related to the investigational product. All adverse events reported spontaneously by the subject or observed by the investigator or her staff will be recorded.

As the additional risk of participants to this study as compared to non-participants only includes additional blood samples and nasal MLF samples. Potential Adverse Events after blood sample collection include hematoma or pain on the venipuncture site. No adverse events are expected from nasal MLF sample collection. Adverse events following venipuncture will be recorded and reported in the annual safety report.

7.2.2 Serious adverse events (SAEs)

A serious adverse event is any untoward medical occurrence or effect that

- results in death;
- is life threatening (at the time of the event);
- requires hospitalisation or prolongation of existing inpatients' hospitalisation;
- results in persistent or significant disability or incapacity;
- is a congenital anomaly or birth defect; or
- any other important medical event that did not result in any of the outcomes listed above due to medical or surgical intervention but could have been based upon appropriate judgement by the investigator.

An elective hospital admission will not be considered as a serious adverse event.

The investigator will report all SAEs that arise during the course of the study to the sponsor without undue delay after obtaining knowledge of the events.

The sponsor will report the SAEs through the web portal *ToetsingOnline* to the accredited METC that approved the protocol, within 7 days of first knowledge for SAEs that result in death or are life threatening followed by a period of maximum of 8 days to complete the initial preliminary report. All other SAEs will be reported within a period of maximum 15 days after the sponsor has first knowledge of the serious adverse events.

7.2.3 Suspected unexpected serious adverse reactions (SUSARs)

Adverse reactions are all untoward and unintended responses to an investigational product related to any dose administered.

Unexpected adverse reactions are SUSARs if the following three conditions are met:

1. the event must be serious (see chapter 8.2.2);
2. there must be a certain degree of probability that the event is a harmful and an undesirable reaction to the medicinal product under investigation, regardless of the administered dose;
3. the adverse reaction must be unexpected, that is to say, the nature and severity of the adverse reaction are not in agreement with the product information as recorded in:
   - Summary of Product Characteristics (SPC) for an authorised medicinal product;
   - Investigator's Brochure for an unauthorised medicinal product.

The sponsor will report expedited the following SUSARs through the web portal *ToetsingOnline* to the METC:

- SUSARs that have arisen in the clinical trial that was assessed by the METC;
- SUSARs that have arisen in other clinical trials of the same sponsor and with the same medicinal product, and that could have consequences for the safety of the subjects involved in the clinical trial that was assessed by the METC.

The remaining SUSARs are recorded in an overview list (line-listing) that will be submitted once every half year to the METC. This line-listing provides an overview of all SUSARs from the study medicine, accompanied by a brief report highlighting the main points of concern.
The expedited reporting of SUSARs through the web portal Eudravigilance or ToetsingOnline is sufficient as notification to the competent authority.

The expedited reporting will occur not later than 15 days after the sponsor has first knowledge of the adverse reactions. For fatal or life-threatening cases the term will be maximal 7 days for a preliminary report with another 8 days for completion of the report.

7.3 Annual safety report

In addition to the expedited reporting of SUSARs, the sponsor will submit, once a year throughout the clinical trial, a safety report to the accredited METC, competent authority, and competent authorities of the concerned Member States.
This safety report consists of:

- a list of all suspected (unexpected or expected) serious adverse reactions, along with an aggregated summary table of all reported serious adverse reactions, ordered by organ system, per study;
- a report concerning the safety of the subjects, consisting of a complete safety analysis and an evaluation of the balance between the efficacy and the harmfulness of the medicine under investigation.

7.4 Follow-up of adverse events

All AEs will be followed until they have abated, or until a stable situation has been reached. Depending on the event, follow up may require additional tests or medical procedures as indicated, and/or referral to the general physician or a medical specialist.

8. STATISTICAL ANALYSIS

Data will be reported quantitatively. All analyses will be performed from the intention-to-treat principle. In the possible case of missing immunological data, the data will be excluded from analysis. Effect estimates will be reported with 95% confidence intervals. A two-sided p-value <0.05 will be considered statistically significant.

8.1 Primary and secondary study parameter(s)

Endpoints will be reported per intervention group as number of endpoints with rates or proportions, means with standard deviation, or medians with inter-quartile range, as appropriate. For comparison of the primary endpoint (IgG titers at day 21 after last COVID-19 vaccination) between intervention groups (groups 1-3) and the reference group (group 4), we will use the Student's t-test or Mann-Whitney U test, as appropriate. The titer difference will be reported for each intervention group with 95% confidence intervals. The 'influenza prior to COVID-19' or 'concomitant influenza/COVID-19' strategies will be considered noninferior to 'COVID-19 alone' if the upper limit of the confidence interval of the titer difference is less than -0.3 on the log10 scale. As secondary analysis of the primary endpoint, we will test for significant differences between the intervention arms using ANOVA, and, in case of significance, evaluate the optimal sequence of vaccination using pairwise testing.

Secondary endpoints (humoral and cellular immune responses and seroconversion) will be assessed by the Student's t-test, Mann-Whitney U-test or chi square test, as appropriate. A two-sided test will be applied and multiple testing correction will be done using the false discovery rate method. A linear mixed model will be used for analyzing dynamic data (repeated measurements of secondary endpoints over time), as it explicitly accounts for the correlations between repeated measurements within each individual.

Exploratory endpoints will be analyzed by systems biology analysis (integration and analysis of omics and immunological data):
The effect of COVID19 and influenza vaccination on circulating markers of inflammation and metabolites on the one hand, and identify which of these biomarkers influence the response to vaccination on the other hand, will be determined. Specifically, multiple layers of omics data (genome-wide gene expression, DNA methylation, chromatin accessibility, metabolites, and proteins) will be associated to immune phenotypes (e.g. titer response index, the main indicator of protection) at each time point. Important pathways involved in the immune response to vaccinations will be identified. Because the cross-omics data are from the same individuals, we will have the unique opportunity to model both additive and interaction effects of different molecules using a linear model (18).

8.2 Other study parameters

No statistical testing for baseline characteristics will be performed.

8.3 Interim analysis

We will not perform interim analyses.

ETHICAL CONSIDERATIONS

8.4 Regulation statement

The study will be conducted according to the principles of the Declaration of Helsinki amended at the General Assembly in October 2013 and in accordance with the Medical Research Involving Human Subjects Act (WMO) and other guidelines, regulations and Acts.
The latest coronavirus restrictions as imposed by the government and the Radboudumc will be strictly adhered to during the study.

8.5 Recruitment and consent

Participants will be approached by open invitation. Written information about the study will be provided. If participants want to participate in the study, a study investigator will discuss the study with them and participants have the opportunity to ask questions. In addition, an independent physician could provide answers to questions participants might have. If participants would like to participate in the study and they meet all the in- and exclusion criteria, informed consent will be signed and participants will be randomized to one of four treatment arms.

8.6 Eligibility of minors or incapacitated subjects

Underaged or incapacitated participants (as judged by the study investigator) will not be eligible for participation in the study.

8.7 Benefits and risks assessment, group relatedness

The beneficial effect of combined or successive influenza- and SARS-CoV-2 vaccination for the individualized participant is unknown, but it is hypothesized that sufficient protection against both targeted viruses will not be hampered. Potential risks to the participants include the side effects of these vaccines, as well as unanticipated new side effects resulting from the combination of both vaccines. In the previously mentioned UK trial, no safety hazards were observed after co-administering influenza vaccine and Comirnaty (19).
Side effects are described in section *investigational product* and further details can be found in the respective SPCs.
Participants will visit the study clinic 3 times for venous blood and MLF samples. Venipuncture imposes no risk to participants. Obtaining mucosal lining fluid by nasosorption may be experienced by participants as 'tickling' and induce sneezing and/or tearing but this is of a temporary nature and without increased risk.

8.8 Compensation for injury

The sponsor/investigator has a liability insurance which is in accordance with article 7 of the WMO. The sponsor (also) has an insurance which is in accordance with the legal requirements in the Netherlands (Article 7 WMO). This insurance provides cover for damage to research subjects through injury or death caused by the study.
The insurance applies to the damage that becomes apparent during the study or within 4 years after the end of the study.

8.9 Incentives

To ensure follow-up and to reward participants for their efforts, a financial incentive of €40,- per visit will be paid after all visits are completed and transportation costs will be reimbursed. In addition, an important incentive will be the access to SARS-CoV-2 booster and influenza vaccines. Damage to participants through injury or death, caused by the study or negligence of study investigators, is not accountable to the principal investigator.

9. ADMINISTRATIVE ASPECTS, MONITORING AND PUBLICATION

9.1 Handling and storage of data and documents

Data will be handled confidentially. A participant identification code list based on non-traceable items will be used to link the data to the participant. The key to the code will be safeguarded by the investigator on a designated location and will be accessible by study password only.
The handling of personal data complies with the EU General Data Protection Regulation and the Dutch Act on Implementation of the General Data Protection Regulation (in Dutch: Uitvoeringswet AVG, UAVG).

The investigator site file and the electronic data from the eCRF will be stored for 15 years. This electronic data form is protected via study-log in credentials and password.
The collection of participants' baseline information will only be stored in the eCRF and cannot be found elsewhere; this is thus considered 'direct entry' and has been discussed with and approved by the local ethics committee.

All information, data, and results that originate from this study may not be disclosed without the written permission of the principal investigator.

Bodily material (from samples) will be stored for 15 years, for which the participants will be asked to grant permission. Study employees will have access to this material. Participants will be asked permission to use their data and samples for future research, and to be invited for future studies.

The local ethics committee will be consulted in case of further research with chance of incidental findings or research unrelated to the current study, in which case it cannot be assumed that the participants' informed consent pertains to this.

9.2 Monitoring and Quality Assurance

We classify this trail as a low-risk study ('laag-risico onderzoek'), with a negligible risk in terms of the risk classification table provided by NFU, because the used medication is already registered, and has been used in previous trials, including in small trials in the same target population.

Monitoring will be performed by the Sponsor. The first monitor visit will take place before inclusion of the first study participant. During this visit the presence and completeness of the relevant Study Files will be checked. Additionally, the quality and competence will be evaluated of the personnel that will perform the informed consent and who will administer the research medication. After inclusion of ten participants at the site, the site will be monitored with a check of the completeness of three informed consent forms and the accuracy of the eligibility criteria. If there are errors in this, more intensive monitoring will take place. Thereafter, monitoring will be performed centrally by checking the completeness of follow-up data from participants with the mobile application. At the closing visit, the presence and completeness of the relevant Study Files will be checked.

9.3 Amendments

A ‘substantial amendment’ is defined as an amendment to the terms of the METC application, or to the protocol or any other supporting documentation, that is likely to affect to a significant degree:

- the safety or physical or mental integrity of the subjects of the trial;
- the scientific value of the trial;
- the conduct or management of the trial; or
- the quality or safety of any intervention used in the trial.

All substantial amendments will be notified to the METC and to the competent authority. Non-substantial amendments (like typing errors or administrative changes) will not be notified to the accredited METC and the competent authority, but will be recorded and filed by the sponsor.

9.4 Annual progress report

The sponsor/investigator will submit a summary of the progress of the trial to the accredited METC once a year. Information will be provided on the date of inclusion of the first subject, numbers of subjects included and numbers of subjects that have completed the trial, serious adverse events/ serious adverse reactions, other problems, and amendments. Since the study we propose runs for one year only, this annual progress report will simultaneously be the end report.

9.5 Temporary halt and (prematurely) end of study report

The sponsor will notify the accredited METC and the competent authority of the end of the study within a period of 90 days. The end of the study is defined as the last participant’s last visit.
The sponsor will notify the METC immediately of a temporary halt of the study, including the reason of such an action.
In case the study is ended prematurely, the sponsor will notify the accredited METC and the competent authority within 15 days, including the reasons for the premature termination.
Within one year after the end of the study, the investigator/sponsor will submit a final study report with the results of the study, including any publications/abstracts of the study, to the accredited METC and the Competent Authority.

9.6 Public disclosure and publication policy

The results of this study will be disclosed unreservedly at the end of the study. The study results, both positive and negative, will be submitted for publication to peer reviewed journals as well as be reported to the Ministry of Health, Welfare and Sport (VWS) and to the National Institute for Public Health and the Environment (RIVM). The trial will be registered in a public trial registry before the first participant is recruited.

10. STRUCTURED RISK ANALYSIS

10.1 Potential issues of concern

An extensive description of the vaccines used in this study can be found in the SPCs.

a. Level of knowledge about mechanism of action

The **Pfizer** corona vaccine is formulated in lipid particles, which enable delivery of RNA into host cells to allow expression of the SARS-CoV-2 S antigen. This elicits an immune response to the S antigen, which protects against COVID-19.

b. Previous exposure of human beings with the test product(s)

Both the SARS-CoV-2 vaccines and the influenza vaccine have been used in vaccination campaigns targeting adults around the world. The influenza vaccine has been used for decades for the prevention of (severe) illness and has a well-established safety profile for this indication. One of the first studies examining mass vaccination with an mRNA vaccine was reported recently, evaluating the effectiveness of the nationwide vaccination program in Israel. It was concluded that the vaccine is effective for a wide range of COVID-19 related outcomes (e.g. infection, hospitalization and death), a finding consistent with that of the randomized trial (16).

c. Can the primary or secondary mechanism be induced in animals and/or in *ex-vivo* human cell material?

No.

d. Selectivity of the mechanism to target tissue in animals and/or human beings

The effect of the vaccination used in this study is restricted to immune cells, with no effects exerted in other tissues.

e. Analysis of potential effect

The effect of different sequences of mRNA vaccination against SARS-CoV-2 and influenza vaccination is unknown. We hypothesize that both vaccines could enhance and prolong the immune response against both viruses. To date, the only study investigating vaccination against SARS-CoV-2 co-administered with an influenza vaccine concerned part of a phase 3 trial of the NVX-CoV2373 subunit vaccine. In this study, co-administration led to more frequent adverse reactions such as tenderness, pain at injection site and fatigue, but no significant difference in AEs and SAEs compared to co-administration with placebo was reported. The study suggested a vaccine efficacy of 87.5% (19).

f. Pharmacokinetic considerations

For the BNT162b2 **Pfizer/BioNTech** vaccine, no traditional pharmacokinetic or biodistribution studies have been performed. Biodistribution studied in rats shows that injections site and the liver are the major sites of distribution. The greatest mean concentration was found remaining in the injection site at each time point. The greatest levels in plasma were observed 1-4 hours post-dose. Over 48 hours, distribution was mainly observed to liver, adrenal glands, spleen and ovaries, with maximum concentrations observed at 8-48 hours post-dose (20).

g. Study population
Our study population is within the licensed target populations of the 2 used vaccines in this study (SARS-CoV-2 vaccine by Pfizer; influenza vaccine).

h. Interaction with other products
For influenza vaccines, no interactions with other products are described. The same is true for the corona vaccines used in this study, although possible interactions are less extensively investigated. Though vaccines could hamper the effects of immunosuppressive drugs, this is considered irrelevant as only volunteers without immunosuppressive medication or conditions will participate in this trial.

i. Predictability of effect
No previous trials have been performed investigating the use of mRNA vaccines *and* influenza vaccines in different sequences. However, the previously mentioned NVX-CoV2373 subunit vaccine in combination with influenza vaccine demonstrated no increase in AEs and SAEs and resulted in an efficacy of 87.5% - providing extra reassurance regarding safety and immunogenicity for our participants.

j. Can effects be managed?
Possible local and systemic side-effects are expected to be minimal and easily manageable.

10.2 Synthesis

In this trial, the the impact of different sequences of combined influenza- and SARS-CoV-2 vaccinations on immunological responses and side effects will be studied. We aim to understand the immunological, molecular and omics mechanisms that mediate the potential interference between influenza and COVID-19 vaccines, and the long-term effects of this interaction. This study is classified as a ‘verwaarloosbaar risico’ (negligible risk) according to the NFU classification. The vaccines used in this study have all been approved by the European Medicine Agency and are given per routine care. No severe side effects are expected. Participants in this trial will undergo collection of venous blood and mucosal lining fluid at 3 time points, both carry a neglectable risk for the participant.

Positive effects of the study could possibly be enhanced and longer protection against COVID-19 and/or influenza.

11. REFERENCES

1. WHO. WHO Coronavirus (COVID-19) Dashboard 2021 [Available from: https://covid19.who.int/.
2. RIVM. Effecten vaccinatie steeds duidelijker zichbaar onder bewonders verpleeghuizen 2021 [updated 17-03-2021; cited 2021 16-04]. Available from: https://www.rivm.nl/nieuws/effecten-vaccinatie-steeds-duidelijker-zichtbaar-onder-bewoners-verpleeghuizen#:~:text=Het%20aantal%20besmettingen%20in%20verpleeghuizen,tegen%20besmettingen%20met%20het%20coronavirus.
3. Widge AT, Rouphael NG, Jackson LA, Anderson EJ, Roberts PC, Makhene M, et al. Durability of Responses after SARS-CoV-2 mRNA-1273 Vaccination. New England Journal of Medicine. 2020;384(1):80-2.
4. Chia WN, Zhu F, Ong SWX, Young BE, Fong S-W, Le Bert N, et al. Dynamics of SARS-CoV-2 neutralising antibody responses and duration of immunity: a longitudinal study. The Lancet Microbe.
5. Kustin T, Harel N, Finkel U, Perchik S, Harari S, Tahor M, et al. Evidence for increased breakthrough rates of SARS-CoV-2 variants of concern in BNT162b2 mRNA vaccinated individuals. medRxiv. 2021:2021.04.06.21254882.
6. Vajo Z, Tamas F, Sinka L, Jankovics I. Safety and immunogenicity of a 2009 pandemic influenza A H1N1 vaccine when administered alone or simultaneously with the seasonal influenza vaccine for the 2009–10 influenza season: a multicentre, randomised controlled trial. The Lancet. 2010;375(9708):49-55.
7. Nolan T, Bernstein DI, Block SL, Hilty M, Keyserling HL, Marchant C, et al. Safety and immunogenicity of concurrent administration of live attenuated influenza vaccine with measles-mumps-rubella and varicella vaccines to infants 12 to 15 months of age. Pediatrics. 2008;121(3):508-16.
8. Lum LCS, Borja-Tabora CF, Breiman RF, Vesikari T, Sablan BP, Chay OM, et al. Influenza vaccine concurrently administered with a combination measles, mumps, and rubella vaccine to young children. Vaccine. 2010;28(6):1566-74.
9. Do VA, Biering-Sørensen S, Fisker AB, Balé C, Rasmussen SM, Christensen LD, et al. Effect of an early dose of measles vaccine on morbidity between 18 weeks and 9 months of age: a randomized, controlled trial in Guinea-Bissau. The Journal of infectious diseases. 2017;215(8):1188-96.
10. Aaby P, Ravn H, Benn CS, Rodrigues A, Samb B, Ibrahim SA, et al. Randomized Trials Comparing Inactivated Vaccine After Medium-or High-titer Measles Vaccine With Standard Titer Measles Vaccine After Inactivated Vaccine. The Pediatric infectious disease journal. 2016;35(11):1232-41.
11. Anderson EJ, Rouphael NG, Widge AT, Jackson LA, Roberts PC, Makhene M, et al. Safety and immunogenicity of SARS-CoV-2 mRNA-1273 vaccine in older adults. New England Journal of Medicine. 2020;383(25):2427-38.
12. Walsh EE, Frenck Jr RW, Falsey AR, Kitchin N, Absalon J, Gurtman A, et al. Safety and immunogenicity of two RNA-based Covid-19 vaccine candidates. New England Journal of Medicine. 2020;383(25):2439-50.
13. Stephenson KE, Le Gars M, Sadoff J, de Groot AM, Heerwegh D, Truyers C, et al. Immunogenicity of the Ad26.COV2.S Vaccine for COVID-19. Jama. 2021;325(15):1535-44.
14. Snape M. Com-COV Protocol. 2021.
15. Snape M. Com-COV2 Protocol. 2021.
16. Dagan N, Barda N, Kepten E, Miron O, Perchik S, Katz MA, et al. BNT162b2 mRNA Covid-19 vaccine in a nationwide mass vaccination setting. New England Journal of Medicine. 2021;384(15):1412-23.
17. Fröberg J, Gillard J, Philipsen R, Lanke K, Rust J, van Tuijl D, et al. Elevated mucosal antibody responses against SARS-CoV-2 are correlated with lower viral load and faster decrease in systemic COVID-19 symptoms. medRxiv. 2021:2021.02.02.21250910.

18. Hemani G, Shakhbazov K, Westra H-J, Esko T, Henders AK, McRae AF, et al. Detection and replication of epistasis influencing transcription in humans. Nature. 2014;508(7495):249-53.
19. Toback S, Galiza E, Cosgrove C, Galloway J, Goodman AL, Swift PA, et al. Safety, Immunogenicity, and Efficacy of a COVID-19 Vaccine (NVX-CoV2373) Co-administered With Seasonal Influenza Vaccines. medRxiv. 2021:2021.06.09.21258556.
20. (CHMP) CfMPfHU. Assessment report Comirnaty. 19-02-2021.
