# Supplementary material for: Timing and sequence of vaccination against COVID-19 and influenza (TACTIC): a single-blind, placebo-controlled randomized clinical trial
Source: Lancet Reg Health Eur. 2023 Apr 12;29:100628. doi: 10.1016/j.lanepe.2023.100628 (PMC10091277; doi:10.1016/j.lanepe.2023.100628)
Supplement: Supplementary Methods S1 [file mmc10.docx]

1. **4E-BP1**
2. **ADA**
3. ARTN
4. AXIN1
5. Beta-NGF
6. CASP-8
7. **CCL11**
8. **CCL19**
9. **CCL20**
10. **CCL23**
11. **CCL25**
12. CCL28
13. **CCL3**
14. **CCL4**
15. **CD244**
16. **CD40**
17. **CD5**
18. CD6
19. **CD8A**
20. CDCP1
21. **CSF-1**
22. **CST5**
23. CX3CL1
24. **CXCL1**
25. **CXCL10**
26. **CXCL11**
27. **CXCL5**
28. **CXCL6**
29. **CXCL9**
30. **DNER**
31. EN-RAGE
32. **FGF-19**
33. FGF-21
34. FGF-23
35. FGF-5
36. **Flt3L**
37. GDNF
38. **HGF**
39. IFN-gamma
40. IL-1 alpha
41. IL10
42. IL-10RA
43. **IL-10RB**
44. **IL-12B**
45. IL13
46. IL-15RA
47. IL-17A
48. IL-17C
49. **IL18**
50. **IL-18R1**
51. IL2
52. IL-20
53. IL-20RA
54. IL-22 RA1
55. IL-24
56. IL-2RB
57. IL33
58. IL4
59. IL5
60. IL6
61. IL7
62. IL8
63. **TGF-beta-1**
64. LIF
65. LIF-R
66. **MCP-1**
67. **MCP-2**
68. MCP-3
69. **MCP-4**
70. **MMP-1**
71. **MMP-10**
72. NRTN
73. NT-3
74. **OPG**
75. **OSM**
76. PD-L1
77. **SCF**
78. SIRT2
79. SLAMF1
80. ST1A1
81. STAMBP
82. TGF-alpha
83. TNF
84. TNFB
85. **TNFRSF9**
86. TNFSF14
87. **TRAIL**
88. **TRANCE**
89. TSLP
90. **TWEAK**
91. **uPA**
92. **VEGFA**

*Supplementary methods 1: 92 proteins of interested measured by the Olink targeted proteomics analysis. The proteins displayed in bold (44 in total) yielded sufficient results for analysis.*
