# Supplementary material for: Timing and sequence of vaccination against COVID-19 and influenza (TACTIC): a single-blind, placebo-controlled randomized clinical trial
Source: Lancet Reg Health Eur. 2023 Apr 12;29:100628. doi: 10.1016/j.lanepe.2023.100628 (PMC10091277; doi:10.1016/j.lanepe.2023.100628)
Supplement: Caption for supplementary material [file mmc11.docx]

**Supplementary material**

*Supplementary methods 1: 92 proteins of interested measured by the Olink targeted proteomics analysis.*

*Supplementary figure 1: Study flow diagram*

*Supplementary figure 2: Geometric mean concentrations (with 95% error bars) of IgA and IgG antibodies against N-protein over the course of the study (a-b)*

*Supplementary figure 3: Concentrations of measured antibodies, induvial data points per group (a-f)*

*Supplementary table 1: Non-inferiority analyses comparing anti-S IgG responses, each group compared to reference group ‘COVID-19 booster only’.*

*Supplementary table 2: Geometric mean concentrations (GMCs) of antibodies at baseline and at 21 days after COVID-19 booster vaccination.*

*Supplementary figure 4: Mucosal antibody responses (a-d) and comparison of anti-S IgG to antibodies found in plasma (e)*

*Supplementary figure 5: Individualized mucosal antibody responses (a-d)*

*Supplementary table 3: Occurrence of reported side-effects within 14 days after first round of vaccination.*

*Supplementary figure 6: The four most commonly reported side-effects within 14 days after first vaccination round, divided by study group: pain at injection site (a); myalgia (b); headache (c); fatigue (d)*

*Supplementary table 4: Relative risks of side-effects (combination group VS reference group)*
